# Near‐Infrared Fluorescent PROTAC Enables Theranostic Imaging and Selective Tau Degradation in Alzheimer's Disease

**DOI:** 10.1002/advs.77545

**Published:** 2026-09-01

**Authors:** Ziwei Ren, Qian Yue, Ning Gao, Qiu‐Lian Hao, Yan Huo, Chuchu Li, Xinyue Leng, Lu‐Lu Sun, Maggie Pui Man Hoi, Hai‐Hao Han, Mingliang Ma, Xu‐Wen Li

**Affiliations:** ^1^ Shanghai Engineering Research Center of Molecular Therapeutics and New Drug Development School of Chemistry and Molecular Engineering East China Normal University Shanghai China; ^2^ Shandong Laboratory of Yantai Drug Discovery Bohai Rim Advanced Research Institute for Drug Discovery Yantai China; ^3^ School of Traditional Chinese Medicine and Health, Nanfang College Guangzhou Guangzhou China; ^4^ Institute of Chinese Medical Sciences, State Key Laboratory of Quality Research in Chinese Medicine, University of Macau Macao China; ^5^ Department of Pharmaceutical Sciences, Faculty of Health Sciences, University of Macau Macau Shandong China; ^6^ State Key Laboratory of Chemical Biology, Molecular Imaging Center Shanghai Institute of Materia Medica Chinese Academy of Sciences Shanghai China; ^7^ Key Laboratory of Brain Functional Genomics Ministry of Education East China Normal University Shanghai China

**Keywords:** Alzheimer's disease, drug lead, near‐infrared fluorescence, PROTAC, P‐Tau protein

## Abstract

The hyperphosphorylated Tau (p‐Tau) protein plays a central role in the pathogenesis of Alzheimer's disease (AD) by driving neurofibrillary tangle formation and neuronal dysfunction. While proteolysis targeting chimeras (PROTACs) offer a promising approach for directly eliminating pathogenic proteins, their real‐time visualization in living systems remains challenging. Here, we report the rational design and synthesis of a series of near‐infrared (NIR) fluorescent Tau‐targeting degraders that integrate theranostic imaging with targeted protein degradation. Among them, compound **D9** emerges as a dual‐functional degrader capable of both high‐contrast fluorescence tracking and potent Tau clearance at 10 nM. Mechanistic investigations indicate that **D9** induces Tau degradation through activation of the ubiquitin–proteasome system (UPS), as confirmed by inhibitor assays. Beyond Tau degradation, **D9** also downregulates amyloid precursor protein (APP) and β‐amyloid (Aβ) expression, suggesting broader neuroprotective effects. In in vivo studies, **D9** significantly promotes p‐Tau clearance and alleviates cognitive deficits in 3 ×Tg‐AD mice. These findings demonstrate that **D9** represents a first‐in‐class NIR fluorescent PROTAC for theranostic imaging and targeted degradation of Tau, providing a powerful platform for visualizing degradation dynamics and developing next‐generation AD therapeutics.

## Graphical abstract

Here, we report a novel NIR fluorescent PROTAC platform that enables real‐time imaging and targeted degradation of Alzheimer's p‐Tau. Our lead compound effectively clears p‐Tau, reduces APP/Aβ, and alleviates cognitive deficits in AD mice. This work demonstrates a theranostic strategy for visualizing degradation and developing next‐generation AD therapies.

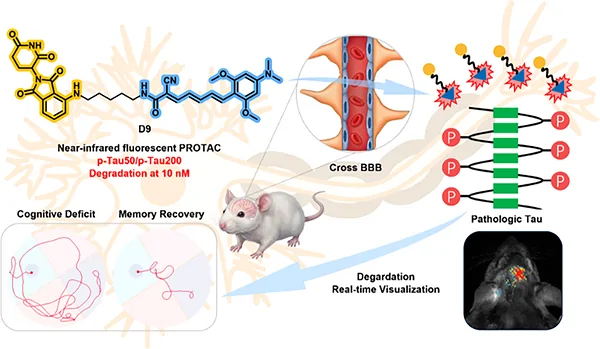

## 1 Introduction

Alzheimer's disease (AD) is a progressive neurodegenerative disorder characterized by memory loss, cognitive dysfunction, and personality change [1]. With an aging global population, its prevalence is increasing rapidly, yet the underlying mechanisms remain elusive and effective disease‐modifying therapies are lacking [2, 3, 4]. Current etiological hypotheses of AD mainly include amyloid plaques, neurofibrillary tangles (NFTs), and intestinal microbes [5, 6, 7, 8, 9, 10]. NFTs, one of the hallmark lesions in AD brains [11, 12], consist mainly of hyperphosphorylated Tau, a microtubule‐associated protein. While physiological Tau contains only a few phosphorylation sites, the aberrant hyperphosphorylation seen in AD‐up to eight sites‐prevents its binding to microtubules, thereby promoting self‐assembly into paired helical filaments and insoluble NFTs [13, 14, 15]. This aggregation disrupts cytoskeletal integrity and neuronal communication, ultimately leading to synaptic loss and neurodegeneration [16, 17]. Therapeutic approaches targeting Tau pathology have largely focused on inhibiting phosphorylation or aggregation, but direct clearance of pathological Tau remains challenging. Recently approved Aβ‐directed antibodies—Donanemab, Lecanemab, and Aducanumab—represent major milestones, yet their clinical benefits are modest and limited to amyloid‐centric mechanisms.

In parallel with therapeutic development, considerable efforts have been devoted to the imaging and diagnosis of Tau pathology in Alzheimer's disease. Existing strategies primarily focus on molecular imaging probes and biosensing platforms that enable visualization of Tau aggregation states or associated biochemical changes. For example, recent advances in biosensors and bioelectronics have enabled sensitive detection of Tau‐related signals, offering valuable tools for early diagnosis and mechanistic studies [18]. In addition, imaging‐oriented theranostic approaches have been explored to monitor Tau pathology progression and therapeutic response [19]. While these methods provide important diagnostic insights, most current Tau imaging strategies are inherently passive, focusing on visualization rather than direct modulation of pathological Tau.

**FIGURE 1:**
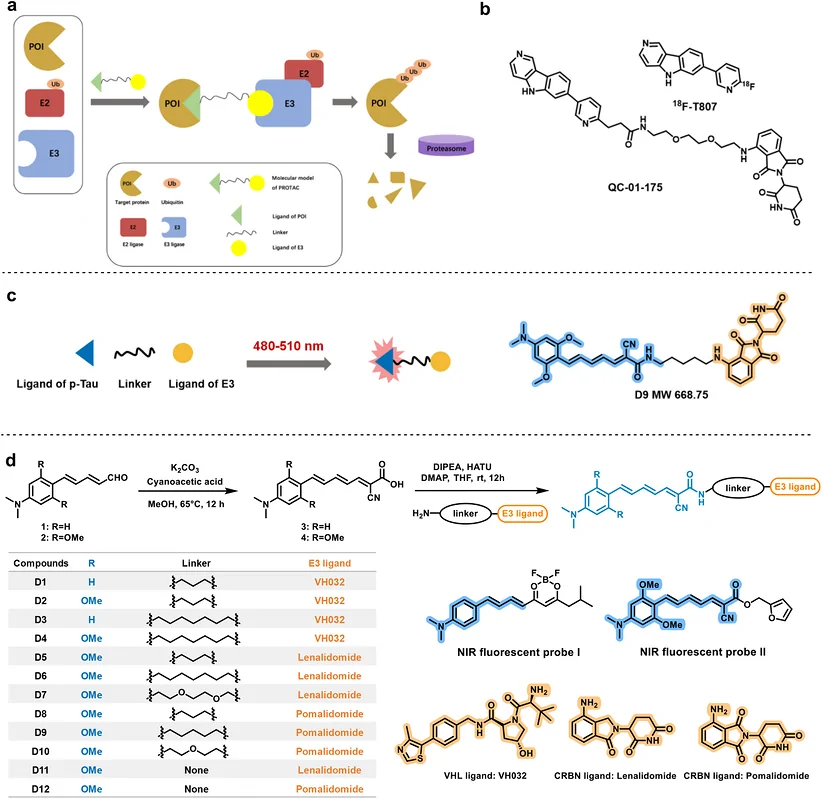
Mechanism and structural design of PROTAC‐based fluorescent Tau degraders inspired by the previous studies. (a) Mechanism of PROTAC technology. (b) Structures of ^18^F‐T807 and QC‐01‐175. (c) Structures of **D9**. p‐Tau ligand (blue), a linker (black), and especially E3 ligand (orange) are contained in the structure. p‐Tau ligand was designed to show NIR fluorescence under 480–510 nm UV irradiation. (d) Design and synthesis of fluorescent Tau degraders.

Proteolysis‐targeting chimeras (PROTACs) have emerged as a transformative modality capable of inducing selective degradation of intracellular proteins via the ubiquitin–proteasome system [20]. A PROTAC molecule typically comprises a ligand recognizing the protein of interest, a ligand recruiting an E3 ubiquitin ligase, and a chemical linker joining the two moieties (Figure 1a) [21, 22, 23, 24]. Several studies have extended this concept to Tau degradation [25, 26]. Chu et al. reported TH006, a peptide‐based PROTAC that efficiently degraded endogenous Tau in cells and AD mouse models [27], while Lu and co‐workers developed Peptide‐1, which recruits the Keap1–Cul3 ligase complex to trigger Tau ubiquitination and proteasomal clearance [28]. Although these pioneering designs validated the feasibility of Tau‐directed degradation, the intrinsic limitations of peptides—poor cell permeability and metabolic instability—hamper their translational potential. More recently, small‐molecule PROTAC degraders of Tau have been reported [29, 30, 31, 32]. Notably, the team led by Gray and Haggarty developed the first Tau‐targeting PROTAC by incorporating the core scaffold of the PET probe ^18^F‐T807. As the radioactive ^18^F moiety required for imaging was not part of this scaffold, the resulting compound exhibited significantly reduced imaging capability (Figure 1b).

To overcome these limitations, we turned to near‐infrared (NIR) fluorescence imaging (650–900 nm), a modality well suited for in vivo applications owing to its deep tissue penetration, low background, and minimal phototoxicity [33]. Guided by this rationale, we designed a series of p‐Tau–targeted PROTACs incorporating an NIR‐emissive scaffold, enabling both targeted degradation and real‐time visualization of Tau dynamics. Remarkably, **D9** (CRBN‐based) achieved efficient removal of both low‐ and high‐molecular‐weight Tau species and displayed a pronounced NIR “turn‐on” effect upon Tau engagement. Subsequent in vivo evaluation confirmed robust degradation efficacy and imaging performance (Figure 1c).

Collectively, our study introduces the first dual‐functional PROTAC integrating targeted protein degradation with fluorescence imaging. This theranostic design not only enables precise monitoring of Tau clearance in real time but also offers a versatile platform for developing next‐generation interventions against Tau‐associated neurodegenerative diseases.

## 2 Results

### 2.1 Design and Synthesis of Tau Degraders

Previous studies identified two near‐infrared (NIR) fluorescent probes, I and II (Figure 1d), as “turn‐on” sensors for Tau aggregates, showing marked fluorescence enhancement upon binding to Tau tangles [34, 35]. Their unique ability to selectively recognize Tau protein provided a suitable scaffold for PROTAC development. Building on this framework, we retained the *N,N*‐dimethylamino group and conjugated olefin chain as the electron donor system and introduced a terminal cyano group as the electron acceptor, which also served as the linkage site for amide coupling to the PROTAC linker.

To optimize degradation efficiency, we systematically varied linker composition and length (alkyl or PEG chains) and coupled them with either VHL‐ or CRBN‐based E3 ligase ligands, generating a focused library of degraders (**D1**‐**D12**) (Figure 1d). The overall synthetic route involved a Wittig extension of the conjugated chain followed by a Knoevenagel condensation with cyanoacetic acid to yield intermediates **3** and **4**, which were subsequently linked to preassembled E3 ligand–linker modules via amide coupling (Figure 1d). Full synthetic details and compound characterization are provided in the Supplementary Information.

### 2.2 Evaluation of Tau Degradation and Fluorescent Characteristics

**FIGURE 2:**
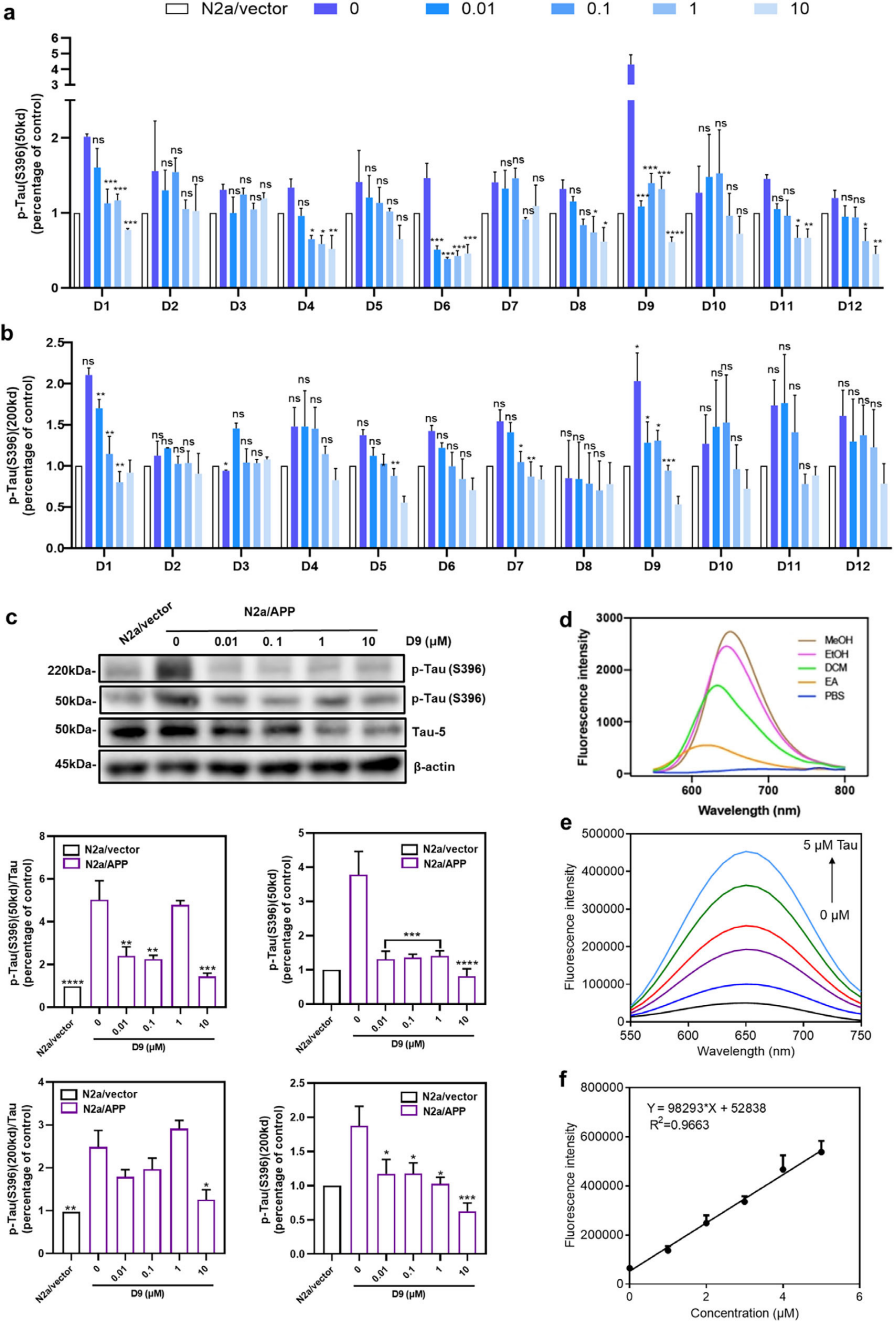
Evaluation of Tau degradation activity and fluorescence characteristics of designed degraders. (a, b) Concentration‐dependent degradation of p‐Tau50 and p‐Tau200 by designed degraders. (c) Western blot analysis and corresponding densitometric quantification of the p‐Tau protein levels in N2a/APP cells treated with **D9** at different concentrations for 24 h. To evaluate both the specific phosphorylation stoichiometry and the absolute net clearance of the pathological Tau burden, p‐Tau levels were normalized to both total Tau (Tau‐5) and the internal control β‐actin. (d) Fluorescence intensity of **D9** in different solvents at the same concentration (25 µm). (e) Fluorescence spectra of **D9** (1 µm) incubated for 5 min in PBS with various concentrations of Tau (0–5 µm), and (**f**) the corresponding linear regression curve. Data are shown as mean ± SD for at least three independent experiments and analyzed by one‐way ANOVA followed by post–hoc Tukey's test. ^*^ *P* ≤ 0.05, ^**^ *P* ≤ 0.01, ^***^ *P* ≤ 0.001 vs. non‐treated N2a/APP group.

The degradation ability of the designed degraders was first evaluated by western blotting (WB) (Figure 2a, b and Figure S49). Several compounds effectively reduced both monomeric p‐Tau (∼50 kDa, p‐Tau50) and aggregated p‐Tau (∼200 kDa, p‐Tau200), confirming the feasibility of using NIR probes as the target‐binding moiety. Structure‐activity relationship (SAR) analysis indicated that both the linker length and the substitution pattern of the probe nucleus had a pronounced impact on activity. For the VHL‐based series, compound **D4** with a long carbon chain linker and methoxy substitution exhibited notable degradation activity toward p‐Tau50. Similarly, in the CRBN‐based series, compound **D6** also showed appreciable activity in reducing p‐Tau50 levels but had a limited effect on aggregated p‐Tau200 (Figure 2a, b). In contrast, compound **D9**, bearing a long carbon chain linker and incorporating the pomalidomide E3 ligand, exhibited the most potent and broad‐spectrum activity among the series, efficiently reducing both p‐Tau50 and p‐Tau200 to normal cellular levels at 10 nm (Figure 2c). Notably, **D9** also features a smaller molecular weight and is therefore predicted to have improved blood–brain barrier (BBB) permeability relative to other series members. By contrast, compounds lacking linkers (**D11**, **D12**) were markedly less effective (Figure 2a, b and Figure S49), underscoring the importance of linker design. Consistent with these results, cytotoxicity assays confirmed that all active degraders showed minimal effects on cell viability below 10 µm (Figure S51). The robust degradation efficiency and favorable structural characteristics distinguish **D9** as the lead compound for subsequent fluorescence property evaluation.

Following the degradation assessment, the fluorescence properties of the lead compound **D9** were further examined (Figure 2d–f). **D9** exhibited a maximum absorption between 480–510 nm (ε = 1.416 × 10^4^ L·mol^−1^·cm^−1^) and an emission peak around 650–700 nm, with a large Stokes shift (>140 nm). The fluorescence intensity of **D9** showed a clear dependence on solvent polarity: strong emission was observed in polar solvents such as methanol and ethanol, weaker signals in less polar solvents including dichloromethane and ethyl acetate, and almost complete quenching in PBS. Remarkably, the fluorescence of **D9** was restored in PBS upon addition of Tau tangles, and the intensity further increased with rising protein concentrations, confirming its “turn‐on” fluorescent behavior in the presence of Tau aggregates and validating its potential as a self‐reporting fluorescent degrader. Notably, additional selectivity studies demonstrated that **D9** exhibited a markedly enhanced fluorescence response toward Tau aggregates compared with Aβ fibrils and representative proteins such as BSA, supporting that the fluorescence turn‐on behavior is primarily driven by specific interactions with Tau rather than nonspecific activation in hydrophobic protein environments (Figure S50).

Together, these results demonstrate that designing fluorescent tau‐targeted PROTACs is a feasible and effective strategy. The efficiency of degradation is strongly influenced by the length of the linker and the pattern of substitution. Among the series, **D9** stood out as the most potent representative, combining high degradation activity with favorable fluorescence properties, which was therefore selected for subsequent mechanistic studies and in vivo evaluation.

### 2.3 Mechanistic Study of Tau Degradation by D9

**FIGURE 3:**
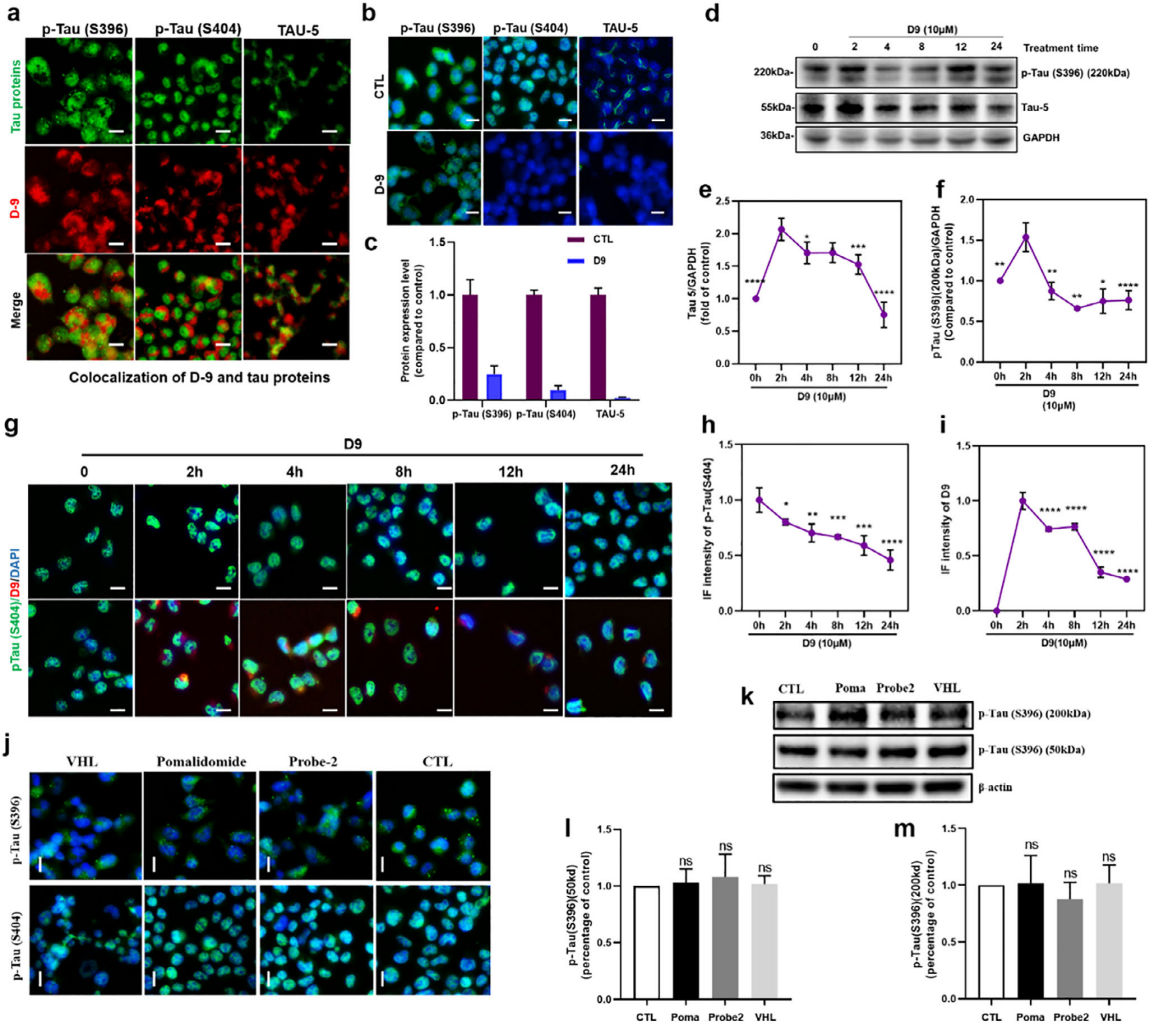
D9 co‐localizes with Tau proteins and reduces intracellular p‐Tau levels; validation of degradation dependence on bifunctional structure. (a, b) **D9** (10 µm, 24 h) was co‐localized with p‐Tau (S396), p‐Tau (S404), and TAU‐5 and reduced those tau species; scale bar = 50 µm. (c) Statistical analysis of fluorescent intensity of tau species after treatment of **D9**. (d) **D9** degrades p‐Tau (S396), Total Tau (TAU) in a time‐dependent manner. (e, f) Statistical analysis of Tau protein levels after treatment with **D9**. (g) Time‐dependent changes in fluorescence intensity of p‐Tau (S396), and TAU‐5 after treatment with **D9**, scale bar = 50 µm. (h, i) Statistical analysis of Tau protein levels after treatment with **D9**. (j) Fluorescence intensity of p‐Tau (S396) and p‐Tau (S404) under treatment with Probe, Pomalidomide, or VHL ligand (10 µm, 24 h). Scale bar = 50 µm. Immunofluorescent intensity was detected using an IN Cell Analyzer 2000 (40 ×, 21°C). Green: Tau protein (Alexa Fluor 488); Blue: nucleus (DAPI). (k) Western blot analysis of p‐Tau (S396) levels after treatment with the individual components. (l, m) Densitometric quantification showing that none of the single components (Probe, Pomalidomide, or VHL) affected the expression levels of p‐Tau (S396). Data represent mean ± SD from at least three independent experiments and were analyzed using *t*‐test. ^*^ *P* ≤ 0.05, ^**^ *P* ≤ 0.01, ^***^ *P* ≤ 0.001, ns > 0.05 vs. non‐treated group.

**D9** was then evaluated for its targeting effect on p‐Tau protein and fluorescence properties at the cellular level (Figure 3). First, the co‐localization of Tau proteins was visualized using an FITC‐fluorescent tracer, while the red fluorescence emitted by the compounds was observed after 24 h of treatment (10 µm for **D9**). Phosphorylated Tau proteins at two distinct phosphorylation sites (S396 and S404), as well as the full‐length Tau (t‐Tau), were individually labeled to assess selectivity. As shown in Figure 3a, **D9** was efficiently taken up by N2a/APP cells and exhibited clear intracellular localization overlapping with Tau signals. **D9**, which showed the most potent degradation ability, exhibited pronounced co‐localization with both p‐Tau (S396) and total Tau (t‐Tau), but weaker affinity for p‐Tau (S404). Quantitative fluorescence analysis (Figure 3b, c) further confirmed these results, indicating that **D9** selectively targets pathological forms of Tau within cells, thereby laying a solid foundation for subsequent in vivo validation. To further quantitatively validate the observed co‐localization patterns, Pearson's correlation coefficients (PCC) and Manders’ overlap coefficients were calculated for **D9** with different Tau species. As summarized in Table S1, **D9** exhibited a higher degree of co‐localization with p‐Tau (S396) (PCC = 0.74) and total Tau (PCC = 0.73), compared to p‐Tau (S404) (PCC = 0.53), consistent with the fluorescence imaging results and supporting the differential selectivity of **D9** toward Tau species.

Furthermore, the dynamic targeting effects of **D9** on p‐Tau (S404) were observed by fluorescence microscopy over a 24 h period (Figure 3g). The compound exhibited a clear time‐dependent degradation of p‐Tau, as the fluorescence intensity of p‐Tau (S404) gradually decreased after 4 h of treatment and continued to decline throughout the 24 h observation (Figure 3h, i). The reduced fluorescence signal at later time points may reflect partial metabolic clearance of **D9** within cells. Overall, the time‐dependent variations in fluorescence intensity of **D9** were consistent with the p‐Tau protein levels determined by Western blotting, confirming the successful visualization of the degradation process. In line with the fluorescence results, Western blot analysis further demonstrated that 4 h treatments with **D9** (Figure 3d–f) significantly decreased the expression of both p‐Tau (S396) and total Tau in N2a/APP cells.

To verify that the degradation effect requires both the E3 ligase and Tau‐binding moieties, N2a/APP cells were treated individually with the probe alone (compound **4**), Pomalidomide, or the VHL ligand at 10 µm for 24 h. Neither the individual probe nor the isolated E3 ligase ligand induced significant changes in p‐Tau (S396) or p‐Tau (S404) expression, as shown by both immunofluorescence and western blot analyses (Figure 3j, k). Quantitative densitometry analysis (Figure 3l, m) further confirmed the absence of degradation activity for any single moiety. These results demonstrate that the complete bifunctional PROTAC structure is indispensable for effective Tau degradation, confirming that physical proximity between Tau and the E3 ligase is essential for proteasomal recruitment and subsequent clearance.

**FIGURE 4:**
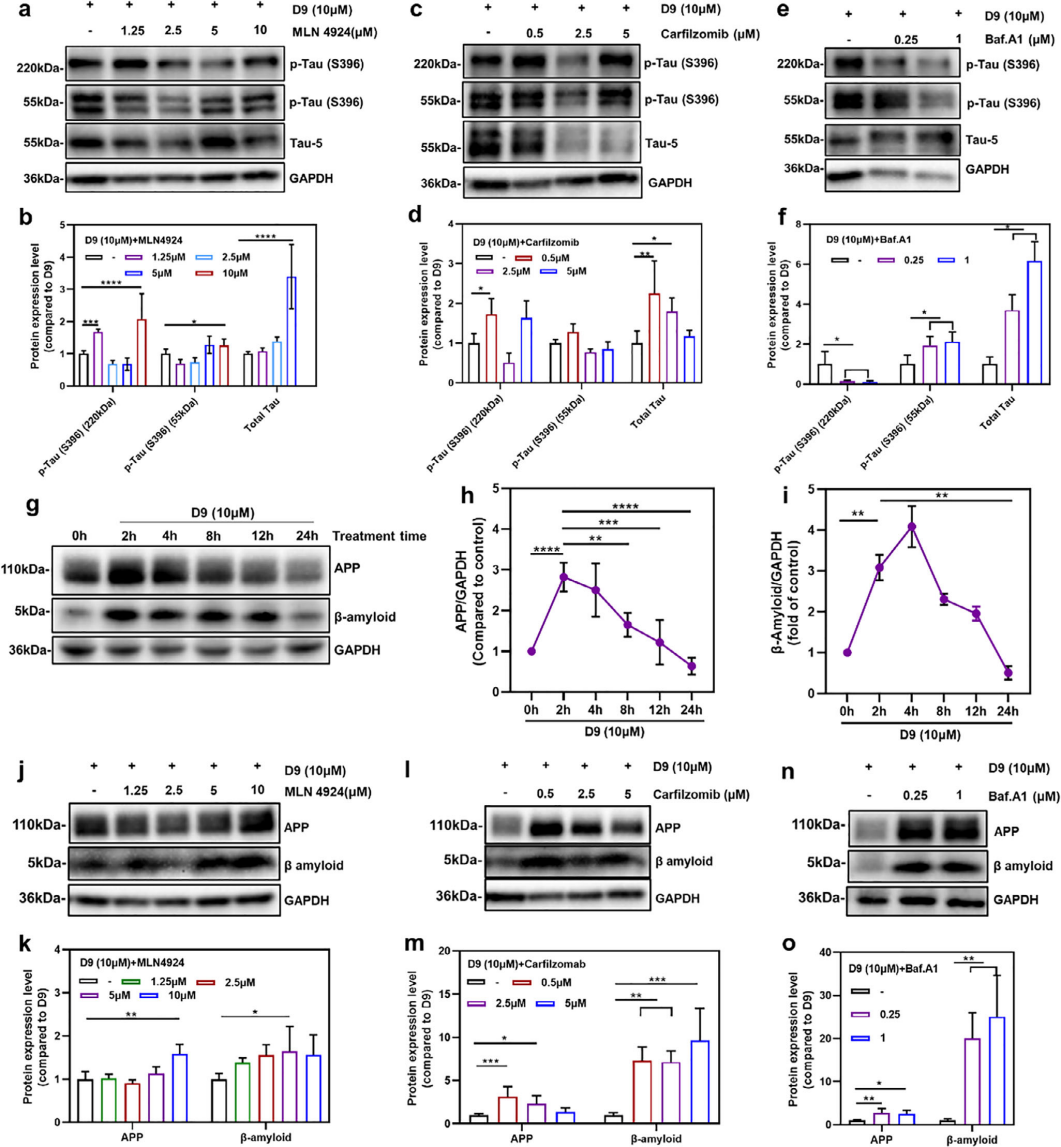
D9 degrades tau through activating UPS and additional effects on β‐amyloid production. (a, b) the NAE inhibitor MLN4924, (c, d) the proteasome inhibitor carfilzomib, or (e, f) the autophagy inhibitor Baf.A1; followed by 18 h treatment with **D9**, for a total of 24 h. P‐Tau (S396) and total Tau (TAU5) were analyzed by western blotting. (g) **D9** induced the degradation of β‐amyloid protein (total) in N2a/APP cells. N2a/APP cells at a serial time period of 2, 4, 6, 8, 12, and 24 h. (h, i) **D9** downregulates the protein expression of APP and β‐amyloid, respectively, in a time‐dependent manner. (j) the NAE inhibitor MLN4924, (l) the proteasome inhibitor carfilzomib, (n) the autophagy inhibitior Baf.A1; followed by 18 h treatment with **D9**, for a total of 24 h. APP and β‐amyloid were analyzed by western blotting. (j, l, n) Representative blots are shown. (k, m, o) Densitometry quantification shows 10 µm MLN4924, 0.5 µm Carfilzomib, and 0.25 µm reverse the degrader effect of **D9**. Data represent mean ± SD from at least three independent experiments and were analyzed using Tukey's test. ^*^ *P* ≤ 0.05, ^**^ *P* ≤ 0.01, ^***^ *P* ≤ 0.001, ns > 0.05 vs. **D9** group.

Then, to mechanistically confirm that the degradation of Tau by **D9** follows the UPS, pathway‐specific inhibitors were employed. We treated N2a/APP cells with **D9** at 10 µm for 18 h, with or without pre‐treatment for 6 h with specific inhibitors, followed by WB analysis of p‐Tau (S396) and total Tau. Pretreatment with MLN4924 (10 µm) (Figure 4a, b) and Carfilzomib (0.5 µm) (Figure 4c, d) rescued the degradation effect of **D9** on p‐Tau and total Tau. The autophagy inhibitor Bafilomycin A1 (Baf.A1) also rescued the degradation of p‐Tau50 (∼55 kDa) and total Tau; however, it promoted the clearance of high‐molecular‐weight p‐Tau200 (∼220 kDa) (Figure 4e, f).

Given that phosphorylated Tau and Aβ are two key pathological hallmarks of AD, we next investigated the effects of our compounds on Aβ. The N2a/APP cell line, which overexpresses amyloid precursor protein (APP), serves as an established cellular model of familial AD. Interestingly, **D9** markedly decreased the protein levels of APP and Aβ in a time‐dependent manner, as shown by western blot analysis (Figure 4g–i). Significant downregulation was observed within 8–12 h and reached maximal reduction at 24 h, indicating that the designed degraders exert broader anti‐amyloid activity in addition to Tau degradation. To verify whether this effect by **D9** occurs through the UPS, N2a/APP cells were pretreated with specific inhibitors prior to **D9** administration. Pretreatment with MLN4924 (10 µm), which blocks E3‐ligase activity by inhibiting the NEDD8‐activating enzyme, and Carfilzomib (0.5–2.5 µm), a proteasome inhibitor, effectively rescued the degradation effects of **D9** on both APP and Aβ (Figure 4j–m). This result suggests that **D9**‐induced clearance of amyloid proteins is associated with activation of the UPS. Furthermore, inhibition of autophagy by Baf.A1 (0.25–1 µm) also attenuated the degradation of APP and Aβ (Figure 4n, o), indicating that autophagy may participate in the clearance process. Notably, while pharmacological inhibition of the UPS significantly impaired **D9**‐mediated degradation, the current data do not directly demonstrate ubiquitination of Tau, and further biochemical validation will be required to confirm this mechanism. In addition, although both UPS and autophagy pathways appear to contribute to **D9**‐induced protein clearance, the precise relationship between these pathways remains to be further elucidated. Taken together, these findings demonstrate that **D9** may activate both UPS‐ and autophagy‐related degradation pathways, leading to efficient removal of multiple AD‐related pathological proteins, including p‐Tau, APP, and Aβ.

### 2.4 Evaluation of Degrader Specificity in 3 ×Tg‐AD Mice

Prior to in vivo imaging and therapeutic evaluation, we first assessed the biosafety of **D9** to address its therapeutic window. Wild‐type mice were administered **D9** (5 mg/kg) via daily repeated dosing for 7 consecutive days. Body weight was monitored throughout the study, followed by serum biochemistry and histological analyses. As shown in Figure S57, no significant body weight loss was observed in **D9**‐treated mice compared with the saline control group. Furthermore, no differences were detected in alanine aminotransferase (ALT), aspartate aminotransferase (AST), and blood urea nitrogen (BUN) levels between groups, and hematoxylin and eosin (H&E) staining revealed no obvious pathological alterations in major organs.

**FIGURE 5:**
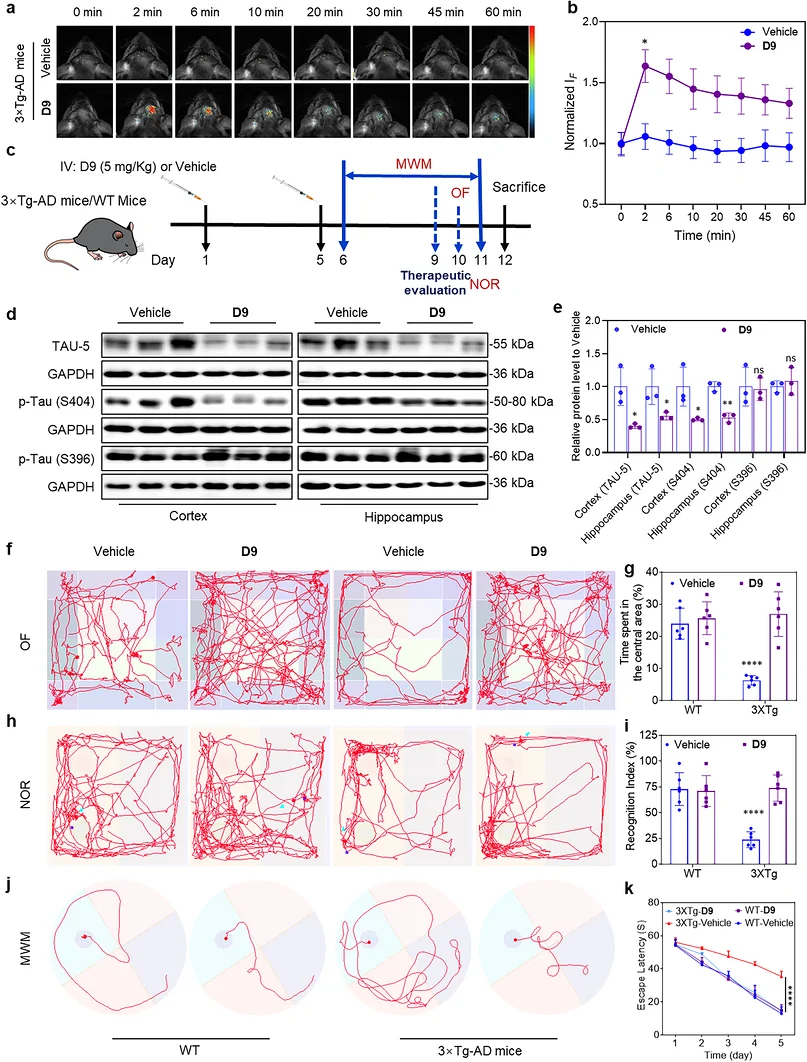
Multi‐dose intravenous (i.v.) injection through the tail vein of D9 promotes Tau clearance and improves cognition in 3×Tg‐AD mice. (a) In vivo NIR fluorescence images of 3×Tg‐AD mice at different time points before and after i.v. injection of **D9** (5 mg/kg). (b) Normalized quantitative analysis of fluorescence signals from **D9** and vehicle at preinjection and 2, 6, 10, 20, 30, 45, and 60 min after i.v. injection. Normalized fluorescence refers to the average radiant efficiency normalized to the Vehicle group at 0 min (set as 1.0). (c) Treatment procedure: **D9** (5 mg/kg) or the vehicle was i.v. injected into 3×Tg‐AD or WT mice every 4 days for a total of 2 times. Following the injection protocol, cognitive behaviors were detected, and additionally, the therapeutic efficacy of **D9** in promoting tau degradation was evaluated by Western blotting to assess the protein levels in hippocampal and cortical tissues. Western blot analysis (d) and quantitative analysis (e) of the expressions of total Tau (TAU‐5), p‐Tau (S404) and p‐Tau (S396) in the hippocampus and cortex of 3×Tg‐AD mice after treating with vehicle or **D9** (dose of 5 mg/kg, via i.v. injection through the tail vein once every 4 days for a total of two times). (f) Representative travel path tracings of 3×Tg‐AD or WT mice in the Open Field (OF) test after **D9** treatment. (g) The time spent in the center area during the OF test. (h) Representative trajectories of 3×Tg‐AD or WT mice in the Novel Object Recognition (NOR) test after **D9** treatment. (i) The discrimination index in the NOR test. (j) Representative swimming paths of 3×Tg‐AD or WT mice in the Morris Water Maze (MWM) test after **D9** treatment. (k) Escape latency of mice in the MWM test during the training phase. Data were shown as mean ± SD (n = 3 in **a‐b**, **d‐e** and n = 6 in **f‐k**), **b** and **f‐k D9** vs. Vehicle in 3×Tg‐AD mice. Data in **b, e, g, i** and **k** were analyzed by t tests. ^*^ *p* < 0.05, ^**^ *p* < 0.01, ^****^ *p* < 0.0001.

We next examined the in vivo NIR fluorescence of **D9** in 3×Tg‐AD and wild‐type (WT) mice following intravenous (i.v.) administration via the tail vein (Figure S52). Within 2 min post‐injection, fluorescence intensity in 3×Tg‐AD mice brains rapidly increased and then gradually declined over 60 min, whereas fluorescence in WT mice remained steadily weak. This dynamic signal enhancement likely reflects elevated Tau expression in the 3×Tg‐AD model and the ability of **D9** to efficiently cross the BBB and bind to Tau aggregates to generate NIR emission. In vivo imaging further confirmed stronger fluorescent signals in **D9**‐treated 3×Tg‐AD mice compared with those in the vehicle group (Figure 5a, b), suggesting effective BBB penetration and target engagement. To further validate the Tau‐targeting capability of **D9** at the tissue level, we performed ex vivo fluorescence co‐localization analysis on brain sections. Frozen brain slices were prepared following **D9** administration and subjected to immunofluorescence staining using an anti‐Tau antibody. As shown in Figure S59, the red fluorescence signal of **D9** exhibited clear spatial overlap with the green fluorescence of Tau staining. Quantitative analysis revealed Pearson correlation coefficients of 0.62 and 0.69 in the hippocampal CA1 and CA3 regions, respectively, confirming that **D9** specifically co‐localizes with Tau in the brain.

Subsequently, we evaluated the therapeutic potential of **D9** in 3×Tg‐AD and WT mice. **D9** (5 mg/kg) was administered twice at four‐day intervals by i.v. injection (Figure 5c). Western blot analysis of hippocampal and cortical tissues revealed significant reductions in total Tau (TAU‐5) and phosphorylated Tau (pS404) following treatment, while pS396 levels remained unchanged (Figure 5d, e and Figure S53). This selectivity suggests preferential degradation of Tau phosphorylated at the S404 site. Notably, this difference may be attributed to the distinct experimental conditions between in vitro and in vivo systems. In vitro, **D9** is directly applied to cells at defined concentrations, whereas in vivo its brain exposure is constrained by the BBB, which may result in suboptimal concentrations that are insufficient to consistently reduce certain Tau species such as p‐Tau (S396). In contrast, p‐Tau (S404) may be more sensitive to lower levels of **D9**, thereby leading to the observed differential efficacy in vivo. For the degradation ability of **D9** on phosphorylated Tau in 3×Tg‐AD mice, the results showed that compared with the untreated group, the fluorescence in **D9**‐administered 3×Tg‐AD mice remained at a lower level within 0–60 min, with the most significant fluorescent difference observed at the 2‐min time point (Figure S54). Ex vivo fluorescent imaging of brains from both groups also exhibited similar trends (Figure S55), indicating the degradation of Tau protein in the **D9**‐treated group. Collectively, these results demonstrate that **D9** injection can effectively clear the total Tau and phosphorylated Tau protein in 3×Tg‐AD mice. To further substantiate the effective clearance of pathological tau, we performed immunofluorescence analysis on brain frozen sections using an antibody against p‐Tau (S404). As shown in Figure S56, compared with the 3×Tg‐AD model group, **D9**‐treated 3×Tg‐AD mice showed significantly reduced fluorescence intensity of p‐Tau (S404) in the hippocampal CA1 and CA3 regions, indicating a marked decrease in the number of neurofibrillary tangles formed by hyperphosphorylated tau. These findings demonstrate that **D9** can effectively clear pathological tau in vivo.

In addition to Tau clearance, we also assessed astrocyte and microglial activation by immunofluorescence staining and Aβ plaque load by Thioflavin S staining. Compared with the WT control group, 3×Tg‐AD model mice exhibited significantly increased fluorescence intensities of Iba1, GFAP, and Thioflavin S, indicative of AD‐related neuroinflammation and Aβ‐associated neurotoxicity. Following **D9** treatment, the fluorescence intensities of Iba1, GFAP and Thioflavin S were markedly reduced compared with the 3×Tg‐AD model group. These results demonstrate that **D9** further alleviates AD‐associated neuroinflammation and neurotoxicity, exerting a comprehensive beneficial therapeutic effect (Figure S58).

To determine whether **D9** treatment alleviates AD‐related behavioral deficits, we conducted a series of cognitive and behavioral tests. The Open Field (OF) test was utilized to investigate the locomotor activity and exploratory behavior of the mice. The exploration trajectories of the mice in this study showed that 3×Tg‐AD mice tended to avoid the central area, spending less time there and traveling shorter distances compared to those in the WT control group, indicating reduced exploratory activity and anxiety‐related behaviors. **D9** treatment remarkably improved the anxiety‐related symptoms of 3×Tg‐AD mice (Figure 5f, g), as evidenced by an increased time spent in the central area.

Mice inherently possess a preference for novel objects, which reflects their cognitive or recognition abilities. In the Novel Object Recognition (NOR) test, mice typically spend more time exploring new objects, indicating that they remember the familiar ones. WT mice showed a greater interest in new objects. In contrast, 3×Tg‐AD mice (vehicle group) had a significantly lower recognition index, suggesting poor recognition memory. Conversely, **D9** treatment significantly enhanced the recognition index of 3×Tg‐AD mice (Figure 5h, i). In the Morris Water Maze (MWM) test, compared to the control group, 3×Tg‐AD mice exhibited spatial learning deficits, while **D9** treatment showed a trend of reduced escape latency, suggesting directed swimming behavior rather than aimless wandering, which is similar to the performance of WT mice or **D9**‐administered WT mice (Figure 5j, k).

Taken together, these results demonstrate that **D9** crosses the BBB, selectively degrades pathological Tau, and ameliorates cognitive and behavioral impairments in 3×Tg‐AD mice. Beyond enabling in situ imaging of Tau dynamics, **D9** thus represents a promising theranostic agent for AD intervention.

## 3 Conclusion

In this study, we developed a series of NIR fluorescent PROTACs that selectively degrade Tau protein, thereby enabling real‐time visualization of the degradation process. To our knowledge, this represents the first integration of PROTAC technology with NIR fluorescence imaging for monitoring Tau clearance. All synthesized compounds effectively degraded both monomeric and aggregated p‐Tau in N2a/APP cells.

Among these, **D9** emerged as the lead molecule, displaying potent degradation of p‐Tau at 10 nm and restoring its levels in pathological cells to those comparable with normal conditions. Mechanistic studies confirmed that **D9**‐induced Tau degradation involves both the ubiquitin–proteasome and autophagy pathways. In addition, **D9** reduced APP and Aβ accumulation, suggesting broader modulation of AD‐related proteostasis.

In vivo studies further demonstrated that **D9** crosses the BBB, promotes p‐Tau clearance, and ameliorates cognitive and behavioral deficits in 3×Tg‐AD mice. Collectively, these results establish **D9** as a dual‐functional theranostic agent capable of both visualizing and eliminating pathological Tau. Beyond providing a powerful tool for studying Tau dynamics, this work also opens a promising avenue for developing next‐generation therapeutics targeting neurodegenerative disorders such as Alzheimer's disease.

## 4 Experimental Section

### 4.1 Chemistry

All solvents and reagents were used from commercial sources without further purification. Flash column chromatography was performed using silica gel from Qingdao Haiyang. ^1^H‐NMR and ^13^C‐NMR date were measured by Bruker‐Ascend‐400 MHz nuclear magnetic resonance instrument. The high‐resolution mass spectrometry data was measured by the Bruker microTOF‐II high‐resolution mass spectrometer. The purity data was mainly determined by Thermo Scientific UltiMate 3000 and Alliance e2695‐2998. Protocols for the synthesis of **D1‐D12** and intermediates are detailed in the Supplementary Information.

### 4.2 Cell Culture and Compound Treatment

N2a cells were cultured in DMEM supplemented with 1% penicillin/streptomycin and 10% FBS. N2a/APP cell line was developed by Jiahong Lu's team in University of Macau. It was transiently transfected with human APPSwe/Ind (APP 695 Swedish/Indiana mutation) (30145, Addgene) plasmids with empty vectors as the control. The stable expression of human APPSwe/Ind gene was induced by additional supplementation with geneticin (G418). (11811023, Gibco)

### 4.3 Neuronal Viability Assay

N2a/APP cells were trypsinized and seeded in 96‐well plates. After 24 h incubation, cells were treated with serial concentrations of compounds (**D1‐D12**) (0.2–50 µm) for 24 h. Then the medium was discarded off and cells were incubated for 4 h at 37°C in 0.5 mg/ml MTT solution (3‐(4,5‐dimethylthiazol‐2‐yl)‐2,5‐diphenyltetrazolium bromide). The living cells could reduce MTT into insoluble. Then the violet formazan crystals in intact cells were then dissolved by 100 µL DMSO. The absorbance was measured at a wavelength of 570 nm by a SpectraMaxR M5 Multi‐Mode Microscope Readers. Cell viability was expressed as a percentage of the vehicle control.

### 4.4 Flow Cytometry

N2a/APP cells were seeded in 24‐well plates. To figure out the retention of compounds in the cytoplasm, the intracellular level of compounds was measured by flow cytometry. Cells were incubated with potential chemical candidates for different time durations from 1 to 24 h. After treatment with chemical candidates, cells were trypsinized and then washed with PBS for three times and discarded by centrifugation. Cells were finally resuspended with PBS and then the intracellular fluorescent level was measured by Flow cytometry (Beckman Coulter Cytoflex) and was analyzed by the software (FlowJo v10.6.2).

### 4.5 Molecular Fluorescence Experimental Methods

1.00 mm stock solution of the compound was prepared for use, and the solvent was DMSO. Sample configuration method for UV detection: take 100.00 µL of the stock solution and dilute the volume to 4.00 mL with PBS to obtain a sample with a concentration of 25 µm for UV maximum absorption detection. Fluorescence detection sample configuration method: take 4.00 µL of the stock solution and dilute the volume to 4.00 mL with PBS to obtain a 1 µm concentration sample for fluorescence maximum emission wavelength detection.

### 4.6 Immunofluorescence Assay

N2a/APP cells were seeded on a 96‐well plate. Potential chemical candidates were applied to treat N2a/APP cells. In this study, we respectively evaluated the effect of chemical candidates on downregulating the level of phosphorylated Tau (p‐Tau) after 24 h treatment and the time‐dependent manner (0, 2, 4, 8, 12, and 24 h) of certain candidate (**D9**) that showed better activities. After treatment with candidates, cells were washed with PBS and fixed in 4% paraformaldehyde (PFA) for 20 min. Then, cells were permeabilized with PBS containing 0.3% Triton X‐100 for 20 min on ice. After being washed 3 times with PBS for 5 min. Cells were blocked with 2% BSA in PBS for 1 h at room temperature. Primary antibodies (1:500) were applied in 2% BSA in PBS and incubated overnight at 4°C. Cells were then washed 3 times with PBS for 5 min. Secondary antibodies (1:1000) conjugated to fluorescent probes (Alexa Fluor 488‐conjugated Rabbit Anti‐Goat IgG (H+L) and Alexa Fluor 594‐conjugated Rabbit Anti‐Goat IgG (H+L)) were added and incubated for 1 h at room temperature. Cells were washed with 4×PBS for 5 min per wash and imaged using IN Cell Analyzer 2000 system (General Electric).

### 4.7 Cell Lysis and Western Blot

After treatment of serial concentrations of compounds (**D1‐D12**, VHL ligand, pomalidomide) (0.01–10 µm) for 24 h, cells were then washed with ice‐cool PBS and lysed for 30 min on ice with lysis buffer (RIPA, 1% cocktail inhibitors, 1% PMSF). For brain samples from animals, the hippocampal and cortical tissues were collected from mice treated with **D9** (5 mg/kg, tail vein injection every 4 days for 2 times). Tissues were homogenized on ice for 3 min in RIPA lysis buffer (supplemented with 1:100 protease inhibitor cocktail). Cell lysates were centrifuged at 12 500 × g for 20 min at 4°C. Protein concentration in the supernatants was measured using the bicinchoninic acid (BCA) assay (Pierce, Rockford, IL). Supernatants were electrophoresed on 10% SDS‐PAGE, and transferred to polyvinylidene fluoride (PVDF) membranes, which were then blocked with 5% non‐fat milk. Immunoblot analysis was performed by incubation with primary antibodies (1:1000) at 4°C overnight. After washing, membranes were incubated for 1 h at room temperature with horseradish peroxidase‐conjugated goat anti‐rabbit IgG (1:2000). Proteins were visualized using an advanced enhanced ECL system (GE Healthcare, Little Chalfont, Buckinghamshire, UK). Semi‐quantifications were performed with densitometry analysis by Bio‐Rad Image 5.1 Software.

### 4.8 In Vivo NIR Fluorescence Imaging wof D9 in the Brain

Mice (ethical approval statement for the animal experiments m20250112) were intravenously injected with **D9** (5 mg/kg) or vehicle via the tail vein once every 4 days for a total of 2 administrations, and in vivo imaging was performed at predefined time points. Prior to imaging, all mice were anesthetized with isoflurane in oxygen, and the cranial fur was shaved using a razor. Fluorescence imaging was conducted using an in vivo imaging system (Newton 7.0, Vilber), with an excitation filter at 640 nm and an emission filter range of 650–750 nm.

### 4.9 Open Field (OF) Test

The OF was performed to assess motor activity and anxiety‐like behavior in mice. Each mouse was placed in an empty square arena (40 cm × 40 cm × 40 cm) and allowed to freely explore for 5 min for environmental adaptation. Following adaptation, locomotor and exploratory behaviors were recorded for 7 min. The time spent in the central vs. marginal zones was analyzed using the Ethovision video tracking system.

### 4.10 Novel Object Recognition (NOR) Test

The NOR test was conducted after the OF test. Mice were placed in a square box (40 cm × 40 cm × 40 cm). On the training day, mice were allowed to explore two identical objects (green square blocks, 3 cm side length) for 7 min. After a 2‐h interval, one object was replaced with a novel object (red cylindrical block, 3 cm diameter), and mice were allowed to interact with both the familiar and novel objects for 7 min. Behavioral trajectories were recorded by a camera positioned above the test arena.

### 4.11 Morris Water Maze (MWM) Test

The MWM test was used to evaluate learning and memory abilities in mice. The apparatus consisted of a circular pool (100 cm diameter) filled with water (22 ± 1°C) to a depth of 26 cm, with an escape platform submerged 2 cm below the water surface. The pool was divided into four quadrants using water maze software. Mice were sequentially placed in different quadrants and trained to locate the hidden platform over 6 consecutive days. Swimming paths and escape latencies were recorded using an automated tracking system.

### 4.12 Statistical Analysis

Each experiment was performed at least three independent times. No data were excluded from the analysis, and no data transformation or normalization was applied unless otherwise stated. Data were examined for consistency and the presence of obvious outliers prior to statistical analysis. For comparisons between two independent groups, an unpaired two‐tailed Student's *t*‐test was used. For multiple‐group comparisons, one‐way analysis of variance (ANOVA) was applied, followed by appropriate post hoc tests where applicable. The level of statistical significance was defined as *p* < 0.05. Statistical significance is indicated as follows: ^*^ *p* < 0.05, ^**^ *p* < 0.01, ^***^ *p* < 0.001. Data from cell‐based experiments are presented as mean ± standard error of the mean (SEM), while data from animal‐level experiments are presented as mean ± standard deviation (SD). The sample size (*n*) for each experiment is specified in the corresponding figure legends. All statistical analyses were performed using GraphPad Prism software.

## Author Contributions

The manuscript was written through contributions of all authors. All authors have given approval to the final version of the manuscript.

## Conflicts of Interest

The authors declare no competing financial interests.

## Supporting information




**Supporting File 1**: advs77545‐sup‐0001‐SuppMat.zip.

**Supporting File 2**: advs77545‐sup‐0002‐SuppMat.docx.

## Data Availability

The data that support the findings of this study are available from the corresponding author upon reasonable request.
