# Supplementary material for: Near‐Infrared Fluorescent PROTAC Enables Theranostic Imaging and Selective Tau Degradation in Alzheimer's Disease
Source: Adv Sci (Weinh). 2026 Sep 1:e77545. Online ahead of print. doi: 10.1002/advs.77545 (PMC13534785; doi:10.1002/advs.77545)
Supplement: Supplementary file 1 [file ADVS-9999-e77545-s002.docx]

**Supporting Information**

**Near-infrared fluorescent PROTAC enables theranostic imaging and selective Tau degradation in Alzheimer’s disease**

**Ziwei Ren^1,*^, Qian Yue^3,4,5,*^, Ning Gao^2,6,*^,** **Qiu-Lian Hao^2,*^, Yan Huo^1^, Chuchu Li^1^, Xinyue Leng^4,5^,** **Lu-Lu Sun^2^, Maggie Pui Man Hoi^4,5^✉, Hai-Hao Han^2,6^✉, Mingliang Ma^1,7^✉, Xu-Wen Li^2,6^✉**

^1^ Shanghai Engineering Research Center of Molecular Therapeutics and New Drug Development, School of Chemistry and Molecular Engineering, East China Normal University, Shanghai, 200062, China.

^2^ Shandong Laboratory of Yantai Drug Discovery, Bohai Rim Advanced Research Institute for Drug Discovery, Yantai, Shandong 264117, China.

^3^ School of Traditional Chinese Medicine and Health, Nanfang College Guangzhou, Guangzhou, 510970, China.

^4^ Institute of Chinese Medical Sciences, State Key Laboratory of Quality Research in Chinese Medicine, University of Macau, Macau SAR, 999078, China.

^5^ Department of Pharmaceutical Sciences, Faculty of Health Sciences, University of Macau, Macao SAR 999078, China.

^6^ State Key Laboratory of Chemical Biology, Molecular Imaging Center, Shanghai Institute of Materia Medica, Chinese Academy of Sciences, Shanghai 201203, China.

^7^ Key Laboratory of Brain Functional Genomics, Ministry of Education, East China Normal University, Shanghai, 200062, China.

* These authors contributed equally.

✉ Correspondence to:

- Xu-Wen Li (xwli@simm.ac.cn)
- Mingliang Ma (mlma@brain.ecnu.edu.cn)
- Hai-Hao Han (hanhaihao@simm.ac.cn)
- Maggie Pui Man Hoi (maghoi@um.edu.mo)

**Table of Contents**

[**1.**	**Chemistry**	3](#_Toc212192215)

[**2.**	**Supplementary Experimental Section**	3](#_Toc212192216)

[**3.**	**NMR spectrum**	14](#_Toc212192217)

[**4.**	**Biology**	38](#_Toc212192218)

[**5.**	**References**	43](#_Toc212192219)

1. **Chemistry**

All solvents and reagents were used from commercial sources without further purification. Flash column chromatography was performed using silica gel from Qingdao Haiyang. ^1^H-NMR and ^13^C-NMR date were measured by Bruker-Ascend-400MHz nuclear magnetic resonance instrument. The high-resolution mass spectrometry data was measured by the Bruker microTOF-II high-resolution mass spectrometer. The purity data was mainly determined by Thermo Scientific UltiMate 3000 and Alliance e2695-2998.

2. **Supplementary Experimental Section**

Compounds **1-4** were synthesized according to the literature^1, 2^.

**Scheme 1**

**Reagents and conditions:** (a) Lenalidomide, HATU, DIPEA, DMAP, DMF, 25°C, 12 h; (b) HCl/EtOH, CH_3_OH, 25°C, 8 h.

**Procedure 1:** A solution of linker acid (1.0 mmol) in DMF (8 mL) was treated with DIPEA (4.0 mmol) under stirring. The reaction mixture was cooled to 0 °C in an ice–water bath and stirred for 10 min, followed by the portionwise addition of HATU (1.5 mmol). After stirring for another 10 min, the mixture was allowed to warm slowly to room temperature and stirred for 30 min. Lenalidomide (1.0 mmol) was then added in portions over 20 min, and the reaction was stirred at room temperature for 12 h. Upon completion, water (18 mL) was added, and the mixture was stirred for 10 min and extracted with ethyl acetate (18 mL). The combined organic layers were dried, filtered, and concentrated under reduced pressure. The crude product was purified by column chromatography (dichloromethane/methanol = 60/1) to afford **Lena-1**.

**Procedure 2:** A solution of **Lena-1** (3.0 mmol) in methanol (10 mL) was treated with HCl/EtOH solution (5 mL) and stirred at 25 °C for 8 h. Upon completion, the reaction mixture was concentrated under reduced pressure to afford the crude product **Lena-2**, which was used directly in the next step without further purification.

The synthetic route for the intermediate using pomalidomide as the E3 ligase ligand is the same as the **Scheme 1.**

**Scheme 2**

**Reagents and conditions:** (a) Pomalidomide, DIPEA, DMF, 90°C, 12 h; (b) HCl/EtOH, CH_3_OH, 25°C, 8 h.

**Procedure 3:** A solution of 2-(2,6-dioxopiperidin-3-yl)-4-fluoroisoindoline-1,3-dione (1.0 mmol) in DMF (15 mL) was treated dropwise with DIPEA (2.0 mmol) under stirring, followed by the addition of linker amine (1.1 mmol). The reaction mixture was gradually heated to 90 °C and stirred for 12 h. After completion, the mixture was cooled to room temperature, and water (30 mL) was added with stirring for 10 min. The resulting mixture was extracted with ethyl acetate (3 × 20 mL), and the combined organic layers were dried, filtered, and concentrated under reduced pressure. The residue was purified by column chromatography (dichloromethane/methanol = 60/1) to afford **Poma-1**. Following general **Procedure 2**, the product **Poma-2** was obtained.

**Scheme 3**

**Reagents and conditions:** (a) linker acid, DIPEA, HATU, DMAP, THF, 25°C, 2 h; (c) HCl/EtOH, CH_3_OH, 25°C, 8 h.

**Procedure 4:** A solution of linker acid (1.0 mmol) in tetrahydrofuran (8 mL) was treated with DIPEA (3.0 mmol) and DMAP (1.1 mmol) under stirring at room temperature. The reaction mixture was cooled to 0 °C in an ice–water bath, followed by the portionwise addition of HATU (2.0 mmol). After stirring for 10 min, the mixture was allowed to warm to room temperature and stirred for 30 min. The VHL ligand (1.0 mmol) was then added in three portions, and the reaction was stirred at room temperature for 2 h. Upon completion, the mixture was concentrated under reduced pressure, and water (20 mL) was added. The resulting mixture was extracted with dichloromethane (20 mL), and the combined organic layers were dried, filtered, and evaporated under reduced pressure. The residue was purified by column chromatography (dichloromethane/methanol = 60/1) to afford **VHL-1**. Following general **Procedure 2**, the product **VHL-2** was obtained.

**Scheme 4**

**Procedure 5:** To a solution of **4** (1.0 mmol) in 7 mL of THF, DIPEA (4.0 mmol) and DMAP (1.2 mmol) were added under stirring, and the reaction solution was stirred at 0 °C. After 10 minutes, HATU (2.0 mmol) was added, and the reaction mixture was naturally warmed to room temperature. After stirring for half an hour, **E3 ligand amine** (1.0 mmol) was added in batches, and the mixture was stirred at room temperature for 12 h. After the reaction was completed, 14 mL of water was added to the reaction solution, extracted with 14 mL of ethyl acetate, dried and spin-dried. The residue was purified by column chromatography (dichloromethane/methanol = 70/1) to afford **PROTAC**.

**(2R,4R)-1-((S)-2-(4-((2E,4E,6E)-2-cyano-7-(4-(dimethylamino)phenyl)hepta-2,4,6-trienamido)butanamido)-3,3-dimethylbutanoyl)-4-hydroxy-N-(4-(4-methylthiazol-5-yl)benzyl)pyrrolidine-2-carboxamide (D1)**

^1^H NMR (400 MHz, Chloroform-*d*) *δ* 8.66 (s, 1H), 7.90 (d, *J* = 11.9 Hz, 1H), 7.43 – 7.31 (m, 7H), 7.24 – 7.14 (m, 1H), 7.01 (dd, *J* = 14.1, 10.3 Hz, 1H), 6.88 – 6.60 (m, 5H), 4.74 (t, *J* = 7.9 Hz, 1H), 4.61 – 4.50 (m, 3H), 4.41 – 4.29 (m, 1H), 4.16 (dd, *J* = 11.2 Hz, 1H), 3.99 – 3.85 (m, 1H), 3.63 (dd, *J* = 10.7 Hz, 1H), 3.46 – 3.33 (m, 2H), 3.03 (s, 6H), 2.51 (s, 3H), 2.49 – 2.44 (m, 1H), 2.37 – 2.25 (m, 2H), 2.18 (dd, *J* = 12.7, 8.4 Hz, 2H), 1.91 – 1.81 (m, 2H), 0.96 (s, 9H).

^13^C NMR (100 MHz, Chloroform-*d*) *δ* 173.60, 171.95, 170.83, 161.72, 153.79, 151.35, 150.30, 149.74, 148.48, 142.91, 138.14, 131.64, 131.13, 130.94, 129.53 (2C), 129.27 (2C), 128.14 (2C), 124.23, 122.86, 112.04 (2C), 111.88, 101.86, 70.19, 58.57, 58.10, 56.93, 43.25, 40.17 (2C), 39.97, 35.85, 34.69, 33.60, 26.47 (3C), 24.86, 16.07 (2C).

HRMS (ESI) m/z calcd for C_42_H_52_N_7_O_5_S (M+H)^+^ 766.3730; found, 766.3751.

Purity: 99.5%.

**2R,4R)-1-((S)-2-(4-((2E,4E,6E)-2-cyano-7-(4-(dimethylamino)-2,6-dimethoxyphenyl)hepta-2,4,6-trienamido)butanamido)-3,3-dimethylbutanoyl)-4-hydroxy-N-(4-(4-methylthiazol-5-yl)benzyl)pyrrolidine-2-carboxamide (D2)**

^1^H NMR (400 MHz, Chloroform-*d*) *δ* 8.67 (s, 1H), 7.92 (d, *J* = 12.3 Hz, 1H), 7.42 – 7.30 (m, 7H), 7.17 – 7.02 (m, 2H), 6.74 (dd, *J* = 8.1 Hz, 1H), 6.62 (t, *J* = 13.2 Hz, 1H), 5.82 (s, 2H), 4.81 – 4.73 (m, 1H), 4.63 – 4.47 (m, 3H), 4.33 (dd, *J* = 14.9, 4.8 Hz, 1H), 4.21 (d, *J* = 11.2 Hz, 1H), 3.89 (s, 6H), 3.64 – 3.57 (m, 2H), 3.51 – 3.43 (m, 1H), 3.40 – 3.33 (m, 1H), 3.06 (s, 6H), 2.52 (s, 3H), 2.37 – 2.28 (m, 2H), 2.16 (s, 1H), 1.87 (s, 2H), 1.39 – 1.32 (m, 1H), 0.96 (s, 9H).

^13^C NMR (100 MHz, Chloroform-*d*) *δ* 173.70, 172.07, 170.75, 162.32, 161.02 (2C), 154.52, 153.88, 152.73, 150.30, 148.47, 138.14, 135.75, 131.66, 130.94, 129.55 (2C), 128.18 (2C), 126.11, 122.84, 117.19, 103.86, 99.44, 88.08 (2C), 70.22, 58.42, 58.22, 56.83, 55.50 (2C), 43.27, 40.31 (2C), 39.83, 35.66, 34.52, 33.59, 26.48 (3C), 25.00, 16.09.

HRMS (ESI) m/z calcd for C_44_H_56_N_7_O_7_S (M+H)^+^ 826.3959; found, 826.3962.

Purity: 98.8%.

**(2R,4RS)-1-((SR)-2-(8-((2E,4E,6E)-2-cyano-7-(4-(dimethylamino)phenyl)hepta-2,4,6-trienamido)octanamido)-3,3-dimethylbutanoyl)-4-hydroxy-N-(4-(4-methylthiazol-5-yl)benzyl)pyrrolidine-2-carboxamide (D3)**

^1^H NMR (400 MHz, Chloroform-*d*) *δ* 8.68 (s, 1H), 7.97 – 7.86 (m, 1H), 7.52 – 7.31 (m, 7H), 7.05 – 6.95 (m, 1H), 6.88 – 6.60 (m, 5H), 6.29 – 6.19 (m, 1H), 6.17 – 6.09 (m, 1H), 4.74 (t, 1H), 4.63 – 4.46 (m, 3H), 4.39 – 4.30 (m, 1H), 4.14 (d, *J* = 11.4 Hz, 1H), 3.60 (d, *J* = 11.4 Hz, 1H), 3.41 – 3.30 (m, 2H), 3.04 (s, 6H), 2.52 (s, 3H), 2.26 – 2.10 (m, 3H), 1.64 – 1.52 (m, 4H), 1.41 – 1.21 (m, 8H), 0.93 (s, 9H).

^13^C NMR (150 MHz, CDCl_3_) *δ* 170.71, 164.34, 156.05, 151.47, 150.44, 150.22, 148.63, 143.44, 138.11, 131.78, 131.11, 130.06, 130.03, 129.72, 129.51, 127.96, 124.37, 123.03, 120.22, 118.63, 116.24, 112.25, 103.25, 102.00, 70.68, 60.03, 59.93, 58.62, 57.80, 43.38, 40.36, 36.07, 32.07, 32.05, 29.84, 29.46, 27.35, 25.68, 22.83, 18.57, 16.23, 14.26.

HRMS (ESI) m/z calcd for C_46_H_60_N_7_O_5_S (M+H)^+^ 822.4351; found, 822.4377.

Purity: 99.3%

**(2R,4R)-1-((S)-2-(8-((2E,4E,6E)-2-cyano-7-(4-(dimethylamino)-2,6-dimethoxyphenyl)hepta-2,4,6-trienamido)octanamido)-3,3-dimethylbutanoyl)-4-hydroxy-N-(4-(4-methylthiazol-5-yl)benzyl)pyrrolidine-2-carboxamide (D4)**

^1^H NMR (400 MHz, Chloroform-*d*) *δ* 8.68 (s, 1H), 7.91 (d, *J* = 12.3 Hz, 1H), 7.46 – 7.29 (m, 7H), 7.04 (dd, *J* = 13.9, 9.6 Hz, 1H), 6.62 (t, *J* = 13.2 Hz, 1H), 6.21 – 6.11 (m, 2H), 5.83 (s, 2H), 4.75 (t, *J* = 8.0 Hz, 1H), 4.63 – 4.50 (m, 3H), 4.35 (dd, *J* = 15.0, 5.2 Hz, 1H), 4.15 (d, *J* = 11.4 Hz, 1H), 3.89 (s, 6H), 3.59 (d, *J* = 8.6 Hz, 1H), 3.39 – 3.31 (m, 2H), 3.06 (s, 6H), 2.52 (s, 3H), 2.23 – 2.12 (m, 3H), 1.63 – 1.51 (m, 4H), 1.36 – 1.27 (m, 8H), 0.94 (s, 9H).

^13^C NMR (100 MHz, Chloroform-*d*) *δ* 173.88, 172.02, 170.68, 161.70, 160.97 (2C), 154.09, 153.63, 152.66, 150.29, 148.40, 138.11, 135.52, 131.57, 130.94, 129.54 (2C), 128.12 (2C), 126.16, 122.93, 117.35, 99.67, 88.11 (2C), 70.09, 58.31, 57.49, 56.69, 55.48 (2C), 43.25, 40.32 (2C), 40.18, 36.23, 35.77, 34.70, 29.70, 29.30, 28.67, 26.54, 26.42 (3C), 25.28, 16.05.

HRMS (ESI) m/z calcd for C_48_H_64_N_7_O_7_S (M+H)^+^ 882.4526; found, 882.4588.

Purity: 95.2%

**(2E,4E,6E)-2-cyano-7-(4-(dimethylamino)-2,6-dimethoxyphenyl)-N-(4-((2-(2,6-dioxopiperidin-3-yl)-1-oxoisoindolin-4-yl)amino)-4-oxobutyl)hepta-2,4,6-trienamide (D5)**

^1^H NMR (400 MHz, DMSO-*d_6_*) *δ* 11.03 (s, 1H), 9.83 (s, 1H), 8.24 – 8.12 (m, 1H), 7.82 (d, *J* = 10.9 Hz, 2H), 7.51 (t, 2H), 7.30 – 7.11 (m, 3H), 6.48 (t, *J* = 12.3 Hz, 1H), 5.93 (s, 2H), 5.15 (d, *J* = 12.9 Hz, 1H), 4.38 (dd, *J* = 17.8 Hz, 2H), 3.86 (s, 6H), 3.29 – 3.20 (m, 2H), 3.03 (s, 6H), 2.95 – 2.87 (m, 1H), 2.61 (d, *J* = 16.5 Hz, 1H), 2.43 – 2.33 (m, 3H), 2.05 – 1.98 (m, 1H), 1.82 (t, 2H).

^13^C NMR (100 MHz, DMSO-*d_6_*) *δ* 173.38, 171.57, 171.46, 168.32, 162.00, 160.95 (2C), 153.20, 152.48, 135.29, 134.32, 134.22, 133.13, 129.09, 125.76, 122.52, 119.50, 116.96, 102.95, 101.81, 88.66 (2C), 55.98 (2C), 51.94, 46.91, 33.67 (2C), 31.68, 29.52, 25.54, 23.09, 22.58.

HRMS (ESI) m/z calcd for C_35_H_39_N_6_O_7_ (M+H)^+^ 655.2891; found, 655.2880.

Purity: 95.6%

**8-((2E,4E,6E)-2-cyano-7-(4-(dimethylamino)-2,6-dimethoxyphenyl)hepta-2,4,6-trienamido)-N-(2-(2,6-dioxopiperidin-3-yl)-1-oxoisoindolin-4-yl)octanamide (D6)**

^1^H NMR (400 MHz, DMSO-*d_6_*) *δ* 11.03 (s, 1H), 9.77 (s, 1H), 8.10 – 8.04 (m, 1H), 7.88 – 7.76 (m, 2H), 7.53 – 7.47 (m, 2H), 7.30 – 7.13 (m, 3H), 6.48 (t, *J* = 12.5 Hz, 1H), 5.92 (s, 2H), 5.15 (dd, *J* = 13.3, 4.7 Hz, 1H), 4.44 – 4.29 (m, 2H), 3.86 (s, 6H), 3.20 – 3.11 (m, 3H), 3.03 (s, 6H), 2.96 – 2.86 (m, 1H), 2.40 – 2.31 (m, 3H), 2.07 – 1.99 (m, 1H), 1.65 – 1.57 (m, 2H), 1.51 – 1.43 (m, 2H), 1.36 – 1.28 (m, 6H).

^13^C NMR (100 MHz, DMSO-*d_6_*) *δ* 173.35, 171.85, 171.56, 168.31, 161.79, 160.94 (2C), 153.18, 153.03, 152.33, 135.16, 134.28, 134.16, 133.13, 129.09, 125.79, 122.56, 119.43, 116.95, 107.38, 102.99, 102.00, 88.71 (2C), 55.99 (2C), 52.00, 46.94, 36.27, 31.62 (2C), 30.29, 29.46, 29.10, 28.97, 26.77, 25.54, 23.11, 19.03.

HRMS (ESI) m/z calcd for C_39_H_47_N_6_O_7_ (M+H)^+^ 711.3477; found, 711.3506.

Purity: 97.2%.

**(2E,4E,6E)-2-cyano-7-(4-(dimethylamino)-2,6-dimethoxyphenyl)-N-(2-(2-(2-((2-(2,6-dioxopiperidin-3-yl)-1-oxoisoindolin-4-yl)amino)-2-oxoethoxy)ethoxy)ethyl)h epta-2,4,6-trienamide (D7)**

^1^H NMR (400 MHz, DMSO-*d_6_*) *δ* 11.01 (s, 1H), 9.71 (s, 1H), 8.06 (t, *J* = 5.1 Hz, 1H), 7.81 (d, *J* = 11.6 Hz, 1H), 7.74 (d, *J* = 7.0 Hz, 1H), 7.54 (s, 2H), 7.42 – 7.36 (m, 1H), 7.26 – 7.20 (m, 2H), 6.51 – 6.43 (m, 1H), 5.93 (s, 2H), 5.14 (dd, *J* = 12.7, 4.6 Hz, 1H), 4.46 – 4.30 (m, 3H), 4.14 (s, 2H), 3.86 (s, 6H), 3.69 (t, 2H), 3.65 – 3.60 (m, 2H), 3.55 – 3.49 (m, 2H), 3.03 (s, 6H), 2.41 – 2.32 (m, 2H), 2.18 (t, *J* = 7.4 Hz, 1H), 2.04 – 1.97 (m, 2H).

^13^C NMR (100 MHz, DMSO-*d_6_*) *δ* 173.35, 171.50, 168.86, 168.23, 162.06, 160.97 (2C), 153.39, 153.23, 152.65, 135.41, 135.33, 133.37, 133.20, 129.11, 126.84, 125.75, 122.49, 120.21, 116.92, 102.98, 101.48, 88.68 (2C), 70.80, 70.43, 69.83, 69.16, 55.99 (2C), 46.92, 31.62 (2C), 30.29, 29.48, 23.02, 22.57.

HRMS (ESI) m/z calcd for C_37_H_43_N_6_O_9_ (M+H)^+^ 715.3070; found, 715.3092.

Purity: 99.6%.

**(2E,4E,6E)-2-cyano-7-(4-(dimethylamino)-2,6-dimethoxyphenyl)-N-(3-((2-(2,6-dioxopiperidin-3-yl)-1,3-dioxoisoindolin-4-yl)amino)propyl)hepta-2,4,6-trienamide (D8)**

^1^H NMR (400 MHz, DMSO-*d_6_*) *δ* 11.10 (s, 1H), 8.20 (t, 1H), 7.90 – 7.80 (m, 1H), 7.59 (t, *J* = 7.6 Hz, 1H), 7.22 (s, 3H), 7.14 – 7.08 (m, 1H), 7.06 – 7.00 (m, 1H), 6.75 (t, 1H), 6.58 – 6.44 (m, 1H), 5.93 (s, 1H), 5.13 – 5.01 (m, 1H), 3.87 (s, 6H), 3.69 – 3.57 (m, 1H), 3.30 – 3.24 (m, 2H), 3.19 – 3.11 (m, 1H), 3.04 (s, 6H), 2.92 – 2.83 (m, 1H), 2.66 – 2.54 (m, 2H), 2.11 – 1.98 (m, 1H), 1.82 – 1.71 (m, 2H).

^13^C NMR (100 MHz, DMSO-*d_6_*) *δ* 173.32, 170.61, 169.26, 167.79, 162.12, 160.95 (2C), 153.25, 153.19, 152.60, 146.68, 136.70, 135.31, 132.75, 125.77, 122.53, 117.62, 116.96, 110.84, 109.64, 102.97, 101.67, 88.65 (2C), 55.97 (2C), 49.00, 42.31, 38.71, 37.33, 29.01, 18.53 (2C), 17.17.

HRMS (ESI) m/z calcd for C_34_H_37_N_6_O_7_ (M+H)^+^ 641.2682; found, 641.2724.

Purity: 95.7%

**(2E,4E,6E)-2-cyano-7-(4-(dimethylamino)-2,6-dimethoxyphenyl)-N-(5-((2-(2,6-dioxopiperidin-3-yl)-1,3-dioxoisoindolin-4-yl)amino)pentyl)hepta-2,4,6-trienamide (D9)**

^1^H NMR (400 MHz, DMSO-*d_6_*) *δ* 11.10 (s, 1H), 8.09 (t, *J* = 5.3 Hz, 1H), 7.81 (d, *J* = 11.8 Hz, 1H), 7.58 (t, *J* = 7.6 Hz, 1H), 7.30 – 7.16 (m, 3H), 7.11 (d, *J* = 8.5 Hz, 1H), 7.02 (d, *J* = 6.3 Hz, 1H), 6.57 – 6.43 (m, 2H), 5.93 (s, 2H), 5.05 (dd, *J* = 12.9, 5.0 Hz, 1H), 3.86 (s, 6H), 3.65 – 3.58 (m, 1H), 3.20 – 3.11 (m, 3H), 3.03 (s, 6H), 2.90 – 2.81 (m, 2H), 2.06 – 1.98 (m, 1H), 1.63 – 1.56 (m, 2H), 1.55 – 1.47 (m, 2H), 1.38 – 1.31 (m, 2H).

^13^C NMR (100 MHz, DMSO-*d_6_*) *δ* 173.30, 170.59, 169.41, 167.79, 161.82, 160.93 (2C), 153.18, 153.06, 152.38, 147.58, 146.88, 136.76, 135.18, 132.66, 125.78, 122.56, 117.70, 110.85, 109.47, 102.97, 101.95, 88.69 (2C), 55.98 (2C), 54.05, 48.99, 42.31, 31.62, 30.28, 29.50, 28.87, 22.62, 18.55, 17.19.

HRMS (ESI) m/z calcd for C_36_H_41_N_6_O_7_ (M+H)^+^ 669.3093; found, 669.3037.

Purity: 97.2%.

**(2E,4E,6E)-2-cyano-7-(4-(dimethylamino)-2,6-dimethoxyphenyl)-N-(2-(2-((2-(2,6-dioxopiperidin-3-yl)-1,3-dioxoisoindolin-4-yl)amino)ethoxy)ethyl)hepta-2,4,6-trienamide (D10)**

^1^H NMR (400 MHz, DMSO-*d_6_*) *δ* 11.09 (s, 1H), 8.10 – 8.04 (m, 1H), 7.81 (dd, *J* = 12.0, 2.1 Hz, 1H), 7.58 (t, *J* = 8.0 Hz, 1H), 7.43 – 7.34 (m, 1H), 7.28 – 7.20 (m, 2H), 7.19 – 7.12 (m, 2H), 7.03 (dd, *J* = 7.0, 2.0 Hz, 1H), 6.66 – 6.59 (m, 1H), 6.47 (t, *J* = 12.5 Hz, 1H), 5.93 (s, 2H), 5.04 (dd, *J* = 13.0, 5.5 Hz, 1H), 3.86 (s, 6H), 3.63 (t, *J* = 5.8 Hz, 2H), 3.56 – 3.50 (m, 2H), 3.48 (d, *J* = 5.8 Hz, 2H), 3.03 (s, 6H), 2.93 – 2.82 (m, 2H), 2.58 (d, *J* = 16.7 Hz, 1H), 2.08 – 1.95 (m, 1H), 0.85 (t, *J* = 5.9 Hz, 1H).

^13^C NMR (100 MHz, DMSO-*d_6_*) *δ* 173.27, 170.56, 169.4, 167.77, 162.08, 160.97 (2C), 158.82, 153.22, 146.88, 136.72, 135.36, 132.55, 125.76, 124.80, 122.51, 117.93, 116.89, 111.15, 109.71, 102.99, 101.62, 88.69 (2C), 69.13, 55.99 (2C), 49.02, 42.11, 34.96, 31.62, 30.29 (2C), 18.55, 17.20.

HRMS (ESI) m/z calcd for C_35_H_39_N_6_O_8_ (M+H)*^+^* 671.2808; found, 671.2829.

Purity: 99.9%.

**(2E,4E,6E)-2-cyano-7-(4-(dimethylamino)-2,6-dimethoxyphenyl)-N-(2-(2,6-dioxopiperidin-3-yl)-1-oxoisoindolin-4-yl)hepta-2,4,6-trienamide (D11)**

^1^H NMR (400 MHz, DMSO-*d_6_*) *δ* 10.99 (s, 1H), 10.12 (s, 1H), 8.00 (d, *J* = 11.7 Hz, 1H), 7.65 – 7.53 (m, 3H), 7.35-7.28 (m, 3H), 6.57 (t, *J* = 12.4 Hz, 1H), 5.93 (s, 2H), 5.15 (dd, *J* = 13.5, 5.2 Hz, 1H), 4.41 (d, *J* = 9.6 Hz, 2H), 3.87 (s, 6H), 3.05 (s, 6H), 2.91 – 2.87 (m, 1H), 2.61 – 2.57 (m, 1H), 2.43 – 2.38 (m, 1H), 2.01 – 1.98 (m, 1H).

^13^C NMR (151 MHz, DMSO-*d_6_*) *δ* 174.28, 172.87, 171.02, 167.73, 160.83, 160.69, 154.14, 153.48, 153.01, 135.89, 135.84, 133.28, 132.82, 129.65, 128.62, 127.15, 125.22, 121.90, 120.15, 116.31, 102.61, 100.62, 88.21, 55.56, 51.48, 46.67, 31.20, 22.50.

HRMS (ESI) m/z calcd for C_31_H_32_N_5_O_6_ (M+H)*^+^* 570.2362; found, 570.2353.

Purity: 98.9%.

**(2R,4R)-1-((S)-2-((2E,4E,6E)-2-cyano-7-(4-(dimethylamino)-2,6-dimethoxyphenyl) hepta-2,4,6-trienamido)-3,3-dimethylbutanoyl)-4-hydroxy-N-(4-(4-methylthiazol-5-yl)benzyl)pyrrolidine-2-carboxamide (D12)**

^1^H NMR (400 MHz, Chloroform-*d*) *δ* 8.67 (s, 1H), 7.86 (d, *J* = 12.3 Hz, 1H), 7.48 (t, *J* = 5.4 Hz, 1H), 7.34 (p, *J* = 10.2, 9.6 Hz, 6H), 7.08 (t, 1H), 6.69 – 6.57 (m, 2H), 5.81 (s, 2H), 4.75 (t, *J* = 7.6 Hz, 1H), 4.60 – 4.50 (m, 3H), 4.33 (dd, *J* = 14.9, 4.7 Hz, 1H), 4.09 (d, *J* = 11.3 Hz, 1H), 3.89 (s, 6H), 3.64 (d, *J* = 10.9 Hz, 1H), 3.06 (s, 6H), 2.52 (s, 3H), 2.15 – 2.07 (m, 1H), 1.27 (d, *J* = 12.0 Hz, 2H), 0.97 (s, 9H).

^13^C NMR (100 MHz, Chloroform-*d*) *δ* 171.34, 170.66, 162.50, 161.12, 154.68, 152.86, 150.27, 148.44, 138.06, 136.43, 131.66, 130.87, 129.51, 128.03, 126.05, 122.75, 116.82, 103.88, 98.48, 88.00, 70.23, 58.48, 58.32, 56.62, 55.47, 43.23, 40.28, 35.96, 35.08, 26.39, 16.07.

HRMS (ESI) m/z calcd for C_40_H_49_N_6_O_6_S (M+H)^+^ 741.3459; found, 741.3434.

Purity: 97.6%.

3. **NMR spectrum**

**(2R,4R)-1-((S)-2-(4-((2E,4E,6E)-2-cyano-7-(4-(dimethylamino)phenyl)hepta-2,4,6-trienamido)butanamido)-3,3-dimethylbutanoyl)-4-hydroxy-N-(4-(4-methylthiazol-5-yl)benzyl)pyrrolidine-2-carboxamide (D1)**

**Figure S1.** ^1^H NMR spectrum of **D1**.

**Figure S2.** ^13^C NMR spectrum of **D1**.

**Figure S3.** LC-MS spectrum of **D1**.

**Figure S4.** Purity of **D1**.

**(2R,4R)-1-((S)-2-(4-((2E,4E,6E)-2-cyano-7-(4-(dimethylamino)-2,6-dimethoxyphenyl)hepta-2,4,6-trienamido)butanamido)-3,3-dimethylbutanoyl)-4-hydroxy-N-(4-(4-methylthiazol-5-yl)benzyl)pyrrolidine-2-carboxamide (D2)**

**Figure S5.** ^1^H NMR spectrum of **D2**.

**Figure S6.** ^13^C NMR spectrum of **D2**.

**Figure S7.** LC-MS spectrum of **D2**.

**Figure S8.** Purity of **D2**.

**(2R,4RS)-1-((SR)-2-(8-((2E,4E,6E)-2-cyano-7-(4-(dimethylamino)phenyl)hepta-2,4,6-trienamido)octanamido)-3,3-dimethylbutanoyl)-4-hydroxy-N-(4-(4-methylthiazol-5-yl)benzyl)pyrrolidine-2-carboxamide (D3)**

**Figure S9.** ^1^H NMR spectrum of **D3**.

**Figure S10.** ^13^C NMR spectrum of **D3**.

**Figure S11.** LC-MS spectrum of **D3**.

**Figure S12.** Purity of **D3**.

**(2R,4R)-1-((S)-2-(8-((2E,4E,6E)-2-cyano-7-(4-(dimethylamino)-2,6-dimethoxyphenyl)hepta-2,4,6-trienamido)octanamido)-3,3-dimethylbutanoyl)-4-hydroxy-N-(4-(4-methylthiazol-5-yl)benzyl)pyrrolidine-2-carboxamide (D4)**

**Figure S13.** ^1^H NMR spectrum of **D4**.

**Figure S14.** ^13^C NMR spectrum of **D4**.

**Figure S15.** LC-MS spectrum of **D4**.

**Figure S16.** Purity of **D4**.

**(2E,4E,6E)-2-cyano-7-(4-(dimethylamino)-2,6-dimethoxyphenyl)-N-(4-((2-(2,6-dioxopiperidin-3-yl)-1-oxoisoindolin-4-yl)amino)-4-oxobutyl)hepta-2,4,6-trienamide (D5)**

**Figure S17.** ^1^H NMR spectrum of **D5**.

**Figure S18.** ^13^C NMR spectrum of **D5**.

**Figure S19.** LC-MS spectrum of **D5**.

**Figure S20.** Purity of **D5**.

**8-((2E,4E,6E)-2-cyano-7-(4-(dimethylamino)-2,6-dimethoxyphenyl)hepta-2,4,6-trienamido)-N-(2-(2,6-dioxopiperidin-3-yl)-1-oxoisoindolin-4-yl)octanamide (D6)**

**Figure S21.** ^1^H NMR spectrum of **D6**.

**Figure S22.** ^13^C NMR spectrum of **D6**.

**Figure S23.** LC-MS spectrum of **D6**.

**Figure S24.** Purity of **D6**.

**(2E,4E,6E)-2-cyano-7-(4-(dimethylamino)-2,6-dimethoxyphenyl)-N-(2-(2-(2-((2-(2,6-dioxopiperidin-3-yl)-1-oxoisoindolin-4-yl)amino)-2-oxoethoxy)ethoxy)ethyl)h epta-2,4,6-trienamide (D7)**

**Figure S25.** ^1^H NMR spectrum of **D7**.

**Figure S26.** ^13^C NMR spectrum of **D7**.

**Figure S27.** LC-MS spectrum of **D7**.

**Figure S28.** Purity of **D7**.

**(2E,4E,6E)-2-cyano-7-(4-(dimethylamino)-2,6-dimethoxyphenyl)-N-(3-((2-(2,6-dioxopiperidin-3-yl)-1,3-dioxoisoindolin-4-yl)amino)propyl)hepta-2,4,6-trienamide (D8)**

**Figure S29.** ^1^H NMR spectrum of **D8**.

**Figure S30.** ^13^C NMR spectrum of **D8**.

**Figure S31.** LC-MS spectrum of **D8**.

**Figure S32.** Purity of **D8**.

**(2E,4E,6E)-2-cyano-7-(4-(dimethylamino)-2,6-dimethoxyphenyl)-N-(5-((2-(2,6-dioxopiperidin-3-yl)-1,3-dioxoisoindolin-4-yl)amino)pentyl)hepta-2,4,6-trienamide (D9)**

**Figure S33.** ^1^H NMR spectrum of **D9**.

**Figure S34.** ^13^C NMR spectrum of **D9**.

**Figure S35.** LC-MS spectrum of **D9**.

**Figure S36.** Purity and UV absorption of **D9**.

**(2E,4E,6E)-2-cyano-7-(4-(dimethylamino)-2,6-dimethoxyphenyl)-N-(2-(2-((2-(2,6-dioxopiperidin-3-yl)-1,3-dioxoisoindolin-4-yl)amino)ethoxy)ethyl)hepta-2,4,6-trienamide (D10)**

**Figure S37.** ^1^H NMR spectrum of **D10**.

**Figure S38.** ^13^C NMR spectrum of **D10**.

**Figure S39.** LC-MS spectrum of **D10**.

**Figure S40.** Purity of **D10**.

**(2E,4E,6E)-2-cyano-7-(4-(dimethylamino)-2,6-dimethoxyphenyl)-N-(2-(2,6-dioxopiperidin-3-yl)-1-oxoisoindolin-4-yl)hepta-2,4,6-trienamide (D11)**

**Figure S41.** ^1^H NMR spectrum of **D11**.

**Figure S42.** ^13^C NMR spectrum of **D11**.

**Figure S43.** LC-MS spectrum of **D11**.

**Figure S44.** Purity of **D11**.

**(2R,4R)-1-((S)-2-((2E,4E,6E)-2-cyano-7-(4-(dimethylamino)-2,6-dimethoxyphenyl) hepta-2,4,6-trienamido)-3,3-dimethylbutanoyl)-4-hydroxy-N-(4-(4-methylthiazol-5-yl)benzyl)pyrrolidine-2-carboxamide (D12)**

**Figure S45.** ^1^H NMR spectrum of **D12**.

**Figure S46.** ^13^C NMR spectrum of **D12**.

**Figure S47.** LC-MS spectrum of **D12**.

**Figure S48.** Purity of **D12**.

4. **Biology**

**Figure S49.** The effect of serial compounds on the protein level of phosphorylated tau. Data are shown as mean ± SEM of three independent experiments and analysed by one-way ANOVA followed by post hoc Dunnett’s test. ^*^*P* ≤ 0.1, ^**^*P* ≤ 0.01, ^***^*P* ≤0.001 vs. LPS treated group.

**Figure S50.** Selectivity assessment of **D9** for Tau over other proteins/amyloids. Fluorescence of **D9** was measured in PBS alone or with 1 μM Tau, Aβ fibrils, or BSA.

**Table S1.** Quantitative co-localization analysis of **D9** with Tau species

**Figure S51.** The effect of compounds on cell viability of N2a/APP and N2a cell. Compounds **D1 D2 D3 D4 D5 D6 D7 D8 D9 D10 D11** and **D12** showed almost no cytotoxic effects on N2a/APP cell. Data are shown as mean±SEM of three independent experiments and analyzed by one-way ANOVA followed by Turkey’s test. ^*^*P* ≤ 0.1, ^**^*P* ≤ 0.01, ^***^*P* ≤ 0.001 vs. LPS treated group.

**Figure S52.** (**a**) *In vivo* NIR fluorescence images of 3×Tg-AD and WT mice at different time points (0, 2, 6, 10, 20, 30, 45 and 60 min) after i.v. injection of **D9** (5 mg/kg). (**b**) Normalized quantitative analysis of fluorescence signals from 3×Tg-AD and WT mice at different time points (0, 2, 6, 10, 20, 30, 45 and 60 min) after i.v. injection of **D9** (5 mg/kg). Data were expressed as mean ± SD (n = 3).

**Figure S53.** Western blot analysis (**a**) and quantitative analysis (**b**) of the expressions of total Tau (TAU-5), p-Tau (S404) and p-Tau (S396) in the hippocampus and cortex of wild-type (WT) mice after treating with vehicle or **D9** (dose of 5 mg/kg, via tail vein injection once every 4 days for a total of two times). Data are shown as mean ± SD for at least three independent experiments and analyzed by t-test. ^*^ *p* < 0.05, ^**^ *p* < 0.01, ns > 0.05 vs. vehicle group.

**Figure S54.** (**a**) *In vivo* NIR fluorescence images of untreated 3×Tg-AD mice and **D9**-administered 3×Tg-AD mice at different time points (0, 2, 6, 10, 20, 30, 45 and 60 min) after i.v. injection with **D9** (5 mg/kg). (**b**) Normalized quantitative analysis of fluorescence signals from untreated 3×Tg-AD mice and D9-administered 3×Tg-AD mice at different time points (0, 2, 6, 10, 20, 30, 45 and 60 min) after i.v. injection with **D9** (5 mg/kg). Data were expressed as mean ± SD (n = 3).

**Figure S55.** (**a**) *Ex vivo* NIR fluorescence images and (**b**) normalized quantitative analysis of brains from untreated 3×Tg-AD mice and **D9**-administered 3×Tg-AD mice after i.v. injection with **D9** (5 mg/kg) for 2 min. Data were expressed as mean ± SD (n = 3). ^*^ *p* < 0.05.

**Figure S56.** Immunofluorescence staining of phosphorylated tau (p-Tau, S404) in the hippocampal region of 3×Tg-AD mice in control and **D9** (5 mg/kg, tail vein injection once every 4 days for a total of two times) treatment groups. Scale bar: 100 μm.

**Figure S57**. In vivo biocompatibility assessment of D9 (5 mg/kg) after repeated tail vein injections over 7 days. (a) Body weight changes in WT mice treated with Saline or D9 over 7 days. (b-d) Serum chemistry parameters, including alanine aminotransferase (ALT), aspartate aminotransferase (AST), and blood urea nitrogen (BUN), were quantified in wild-type (WT) mice after 7-day repeated treatment with saline or D9. (e) Representative hematoxylin and eosin (H&E) staining images of major organs (heart, liver, spleen, kidney, lung, brain) from Saline- and D9-treated WT mice. n = 3 per group. Data are represented as mean ± SD, unpaired two-tailed Student's t-test in b, c, and d. ns: not significant. Scale bar: 100 μm.

**Figure S58**. Assessment of neuroinflammation and neurotoxicity after **D9** treatment. Immunofluorescence images of (**a**) Iba1 (a microglial marker) and (**b**) GFAP (an astrocytic marker) in the hippocampal region of WT, vehicle-treated 3×Tg-AD mice, and D9-treated 3×Tg-AD mice. (**c**) Detection of Aβ deposition by Thioflavin S staining in the hippocampal region of WT, vehicle- and **D9**-treated 3×Tg-AD mice. Scale bar: 100 μm.

**Figure S59**. Confocal laser scanning microscopy (CLSM) images of immunofluorescence co-localization of **D9** and Tau (immunostained with an anti-Tau antibody) in the hippocampal region of 3×Tg-AD mice. Scale bar: 100 μm.

5. **References**
1. Park, K.-S. *et al.* A difluoroboron β-diketonate probe shows “turn-on” near-infrared fluorescence specific for tau fibrils. *ACS Chem. Neurosci.* **8**, 2124-2131 (2017).
2. Seo, Y. *et al.* A smart near-infrared fluorescence probe for selective detection of tau fibrils in Alzheimer’s disease. *ACS Chem. Neurosci.* **7**, 1474-1481 (2016).
