# Supplementary material for: Near‐Infrared Fluorescent PROTAC Enables Theranostic Imaging and Selective Tau Degradation in Alzheimer's Disease
Source: Adv Sci (Weinh). 2026 Sep 1:e77545. Online ahead of print. doi: 10.1002/advs.77545 (PMC13534785; doi:10.1002/advs.77545)
Supplement: Supplementary file 2 [file ADVS-9999-e77545-s001.zip › t-tau-0.73.pdf]

Colocalization_of_t-tau-16.tif_versus_t-tau-16-drug.tif_1717764526.pdf

Li - Ch1

256.0 x 256.0

Channel 2
t-tau-16-drug.tif

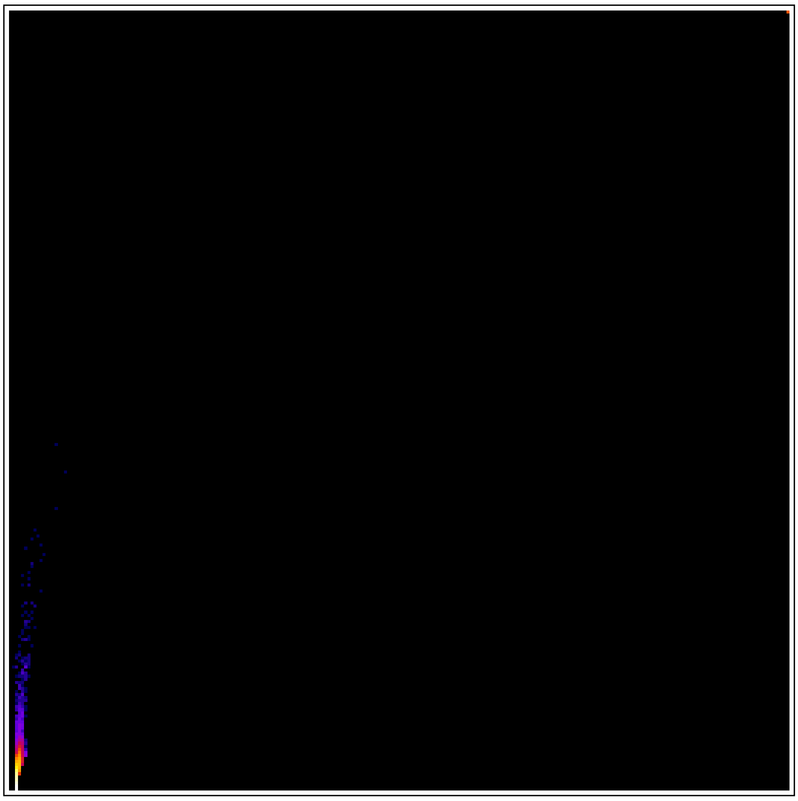

Channel 1
t-tau-16.tif

Li - Ch2

256.0 x 256.0

Channel 2
t-tau-16-drug.tif

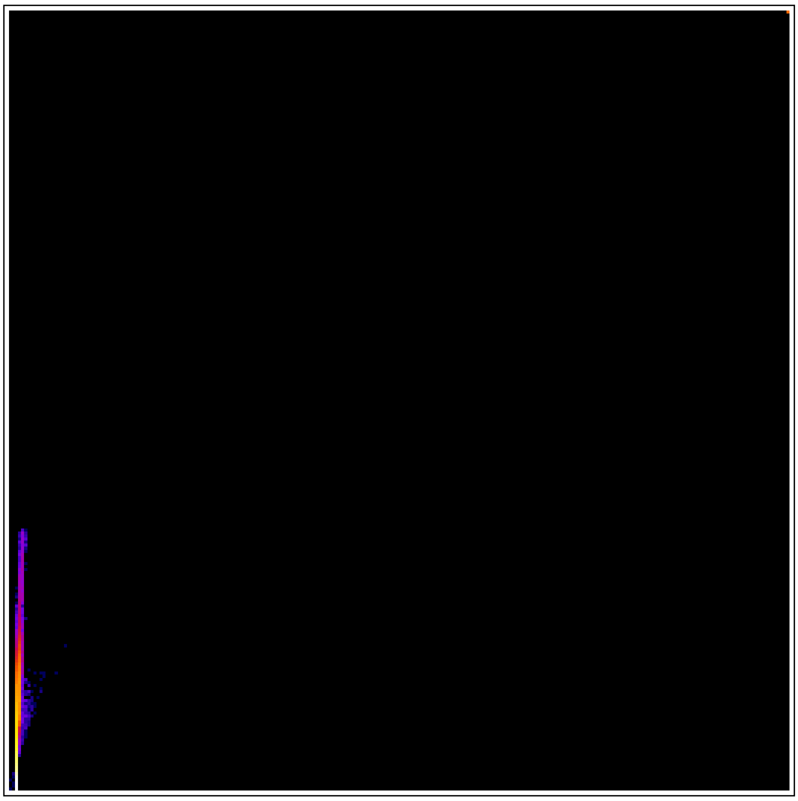

Channel 1
t-tau-16.tif

2D intensity histogram

256.0 x 256.0

Channel 2
t-tau-16-drug.tif

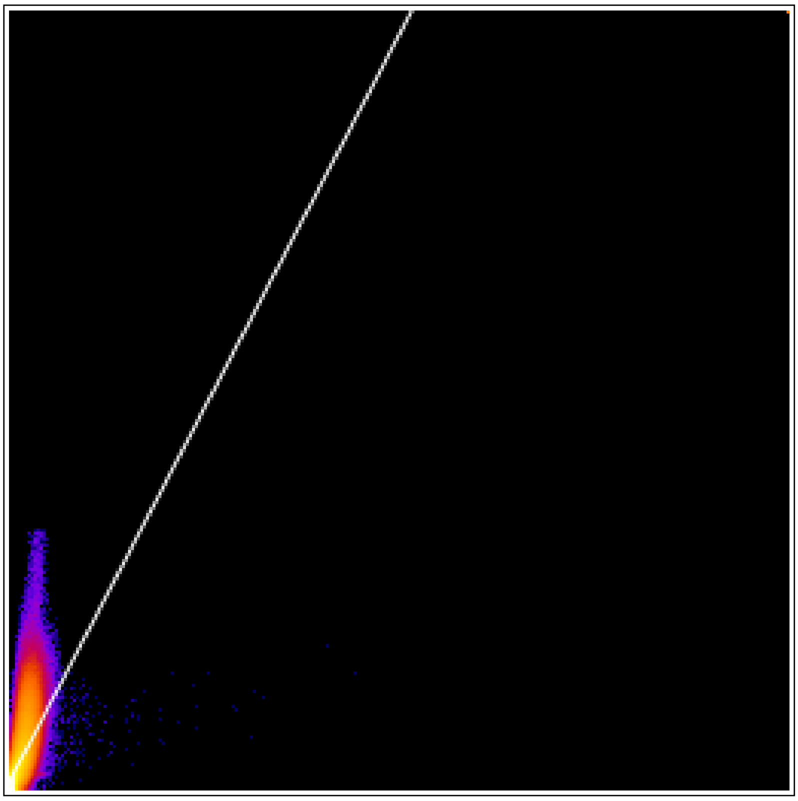

Channel 1
t-tau-16.tif

Smoothed & shuffled channel 1

1024.0 x 1024.0

Channel 2
t-tau-16-drug.tif

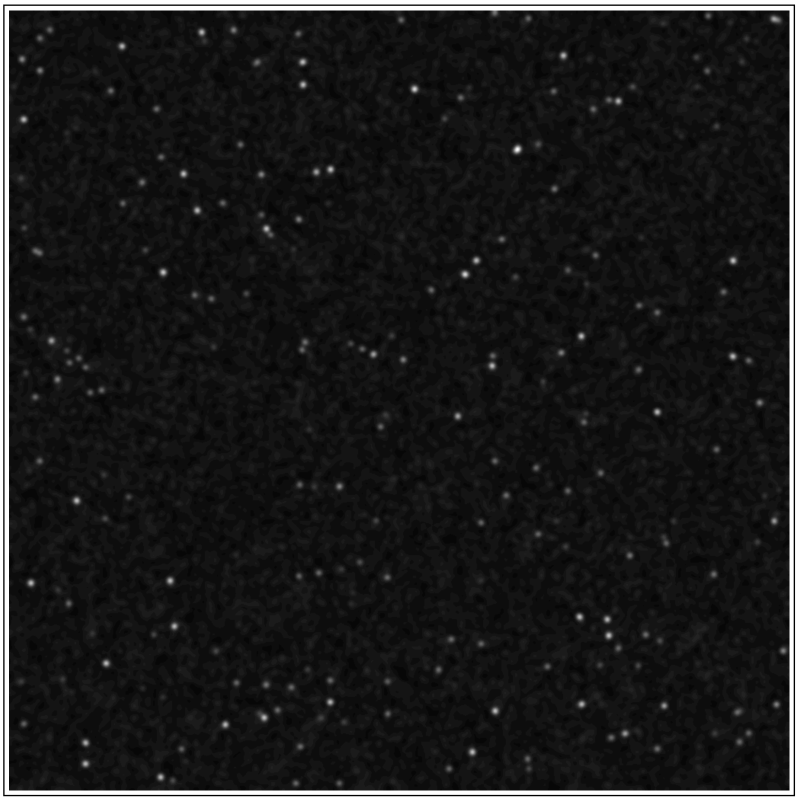

Channel 1
t-tau-16.tif

Channel 1 (Max Projection)

1024.0 x 1024.0

Channel 2
t-tau-16-drug.tif

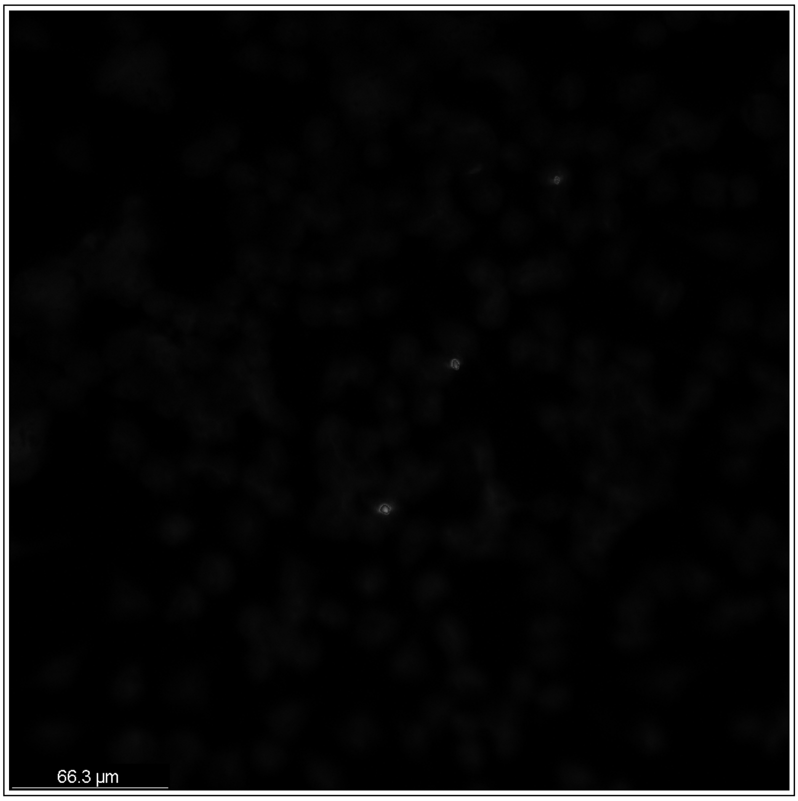

Channel 1
t-tau-16.tif

Channel 2 (Max Projection)
1024.0 x 1024.0

Channel 2
t-tau-16-drug.tif

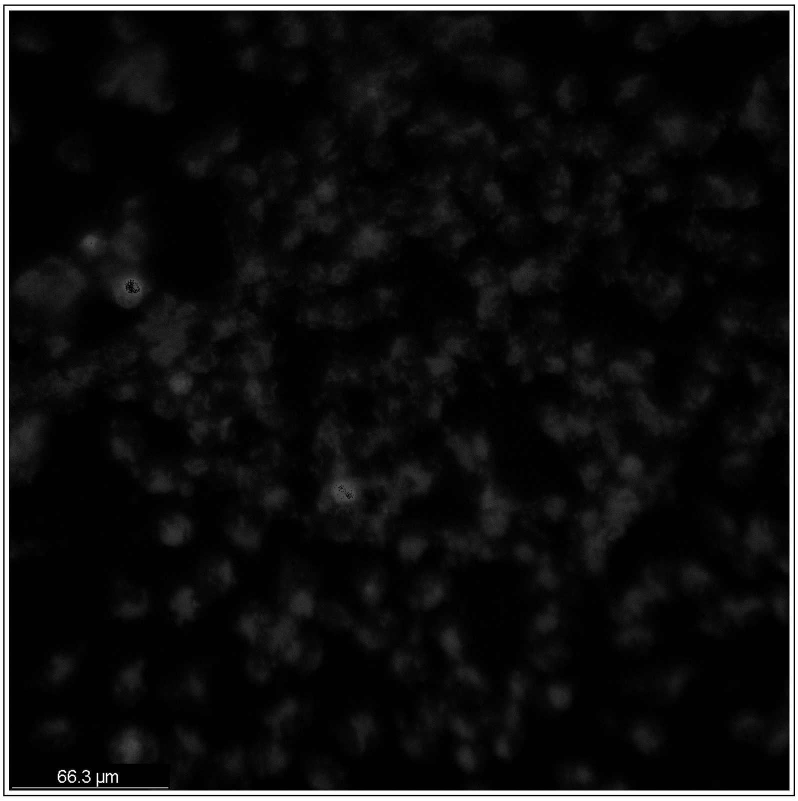

Channel 1
t-tau-16.tif

Warning! y-intercept far from zero - The ratio of the y-intercept of the auto threshold regression line to the mean value of Channel 2 is high. This means the y-intercept is far from zero, implying a significant positive or negative zero offset in the image data intensities. Maybe you should use a ROI. Maybe do a background subtraction in both channels. Make sure you didn't clip off the low intensities to zero. This might not affect Pearson's correlation values very much, but might harm other results.
Warning! Threshold of ch. 1 too high - Too few pixels are taken into account for above-threshold calculations. The threshold is above the channel's mean.
Warning! Threshold of ch. 2 too high - Too few pixels are taken into account for above-threshold calculations. The threshold is above the channel's mean.
Coloc_Job_Name: Colocalization_of_t-tau-16.tif_versus_t-tau-16-drug.tif_1717764526
% zero-zero pixels: 6.79
% saturated ch1 pixels: 0.12
% saturated ch2 pixels: 0.12
Channel 1 Max: 255.000
Channel 2 Max: 255.000
Channel 1 Min: 0.000
Channel 2 Min: 0.000
Channel 1 Mean: 2.705
Channel 2 Mean: 8.521
Channel 1 Integrated (Sum) Intensity: 2836063.000
Channel 2 Integrated (Sum) Intensity: 8935202.000

Mask Type Used: none

Mask ID Used: 1717764526

m (slope): 1.93

b (y-intercept): 3.31

b to y-mean ratio: 0.39

Ch1 Max Threshold: 24.00

Ch2 Max Threshold: 49.00

Threshold regression: Costes

Pearson's R value (no threshold): 0.73

Pearson's R value (below threshold): 0.70

Pearson's R value (above threshold): 0.94

Li's ICQ value: 0.350

Spearman's rank correlation value: 0.77531754

Spearman's correlation t-statistic: 1257.0615

t-statistic degrees of freedom: 1048574.000

Manders' M1 (Above zero intensity of Ch2): 0.973

Manders' M2 (Above zero intensity of Ch1): 0.948

Manders' tM1 (Above autothreshold of Ch2): 0.063

Manders' tM2 (Above autothreshold of Ch1): 0.019

Kendall's Tau-b rank correlation value: 0.6197

Costes P-Value: 1.00

Costes Shuffled Mean: -0.01

Costes Shuffled Std.D.: 0.01

Ratio of rand. Pearsons >= actual Pearsons value : 0.00
