# Supplementary material for: Near‐Infrared Fluorescent PROTAC Enables Theranostic Imaging and Selective Tau Degradation in Alzheimer's Disease
Source: Adv Sci (Weinh). 2026 Sep 1:e77545. Online ahead of print. doi: 10.1002/advs.77545 (PMC13534785; doi:10.1002/advs.77545)
Supplement: Supplementary file 2 [file ADVS-9999-e77545-s001.zip › ptau396-0.74.pdf]

Colocalization_of_ptau396-16.tif_versus_ptau396-16-drug.tif_110273625.pdf

Li - Ch1

256.0 x 256.0

Channel 2
ptau396-16-drug.tif

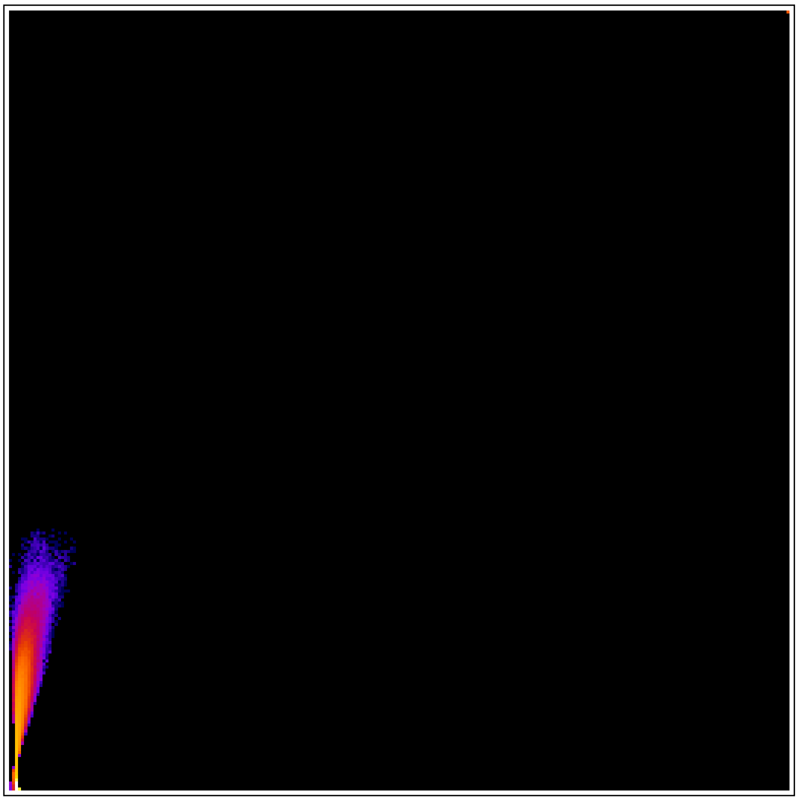

Channel 1
ptau396-16.tif

Li - Ch2

256.0 x 256.0

Channel 2
ptau396-16-drug.tif

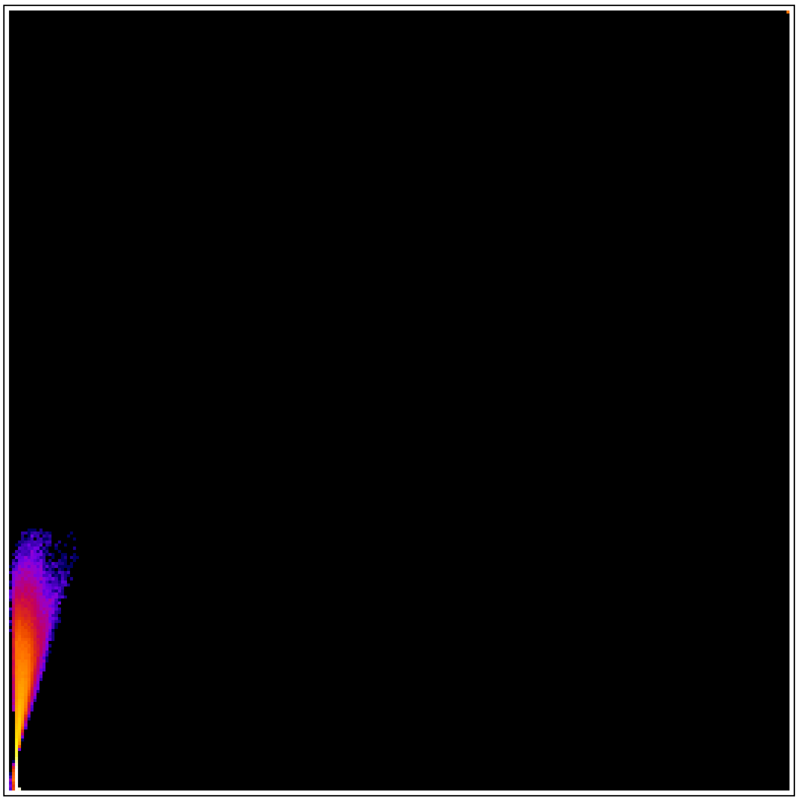

Channel 1
ptau396-16.tif

2D intensity histogram

256.0 x 256.0

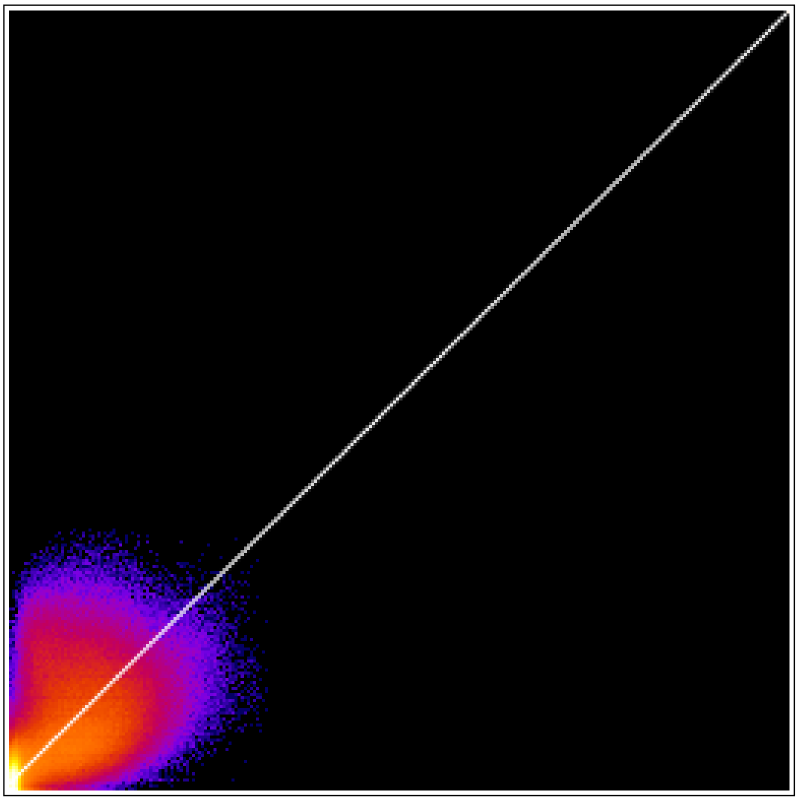

Smoothed & shuffled channel 1

1024.0 x 1024.0

Channel 2
ptau396-16-drug.tif

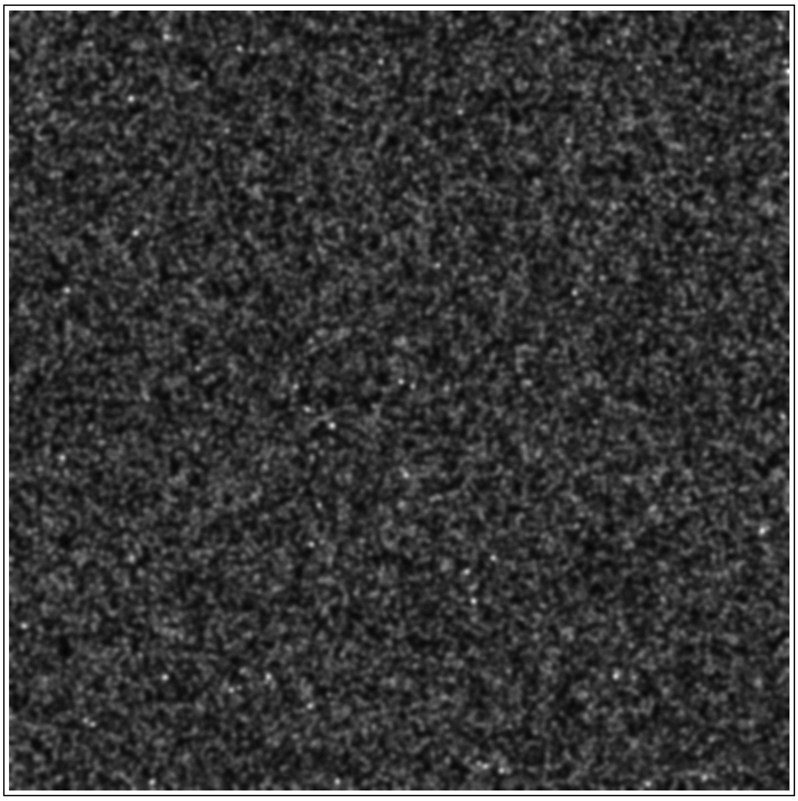

Channel 1
ptau396-16.tif

Channel 1 (Max Projection)

1024.0 x 1024.0

Channel 2
ptau396-16-drug.tif

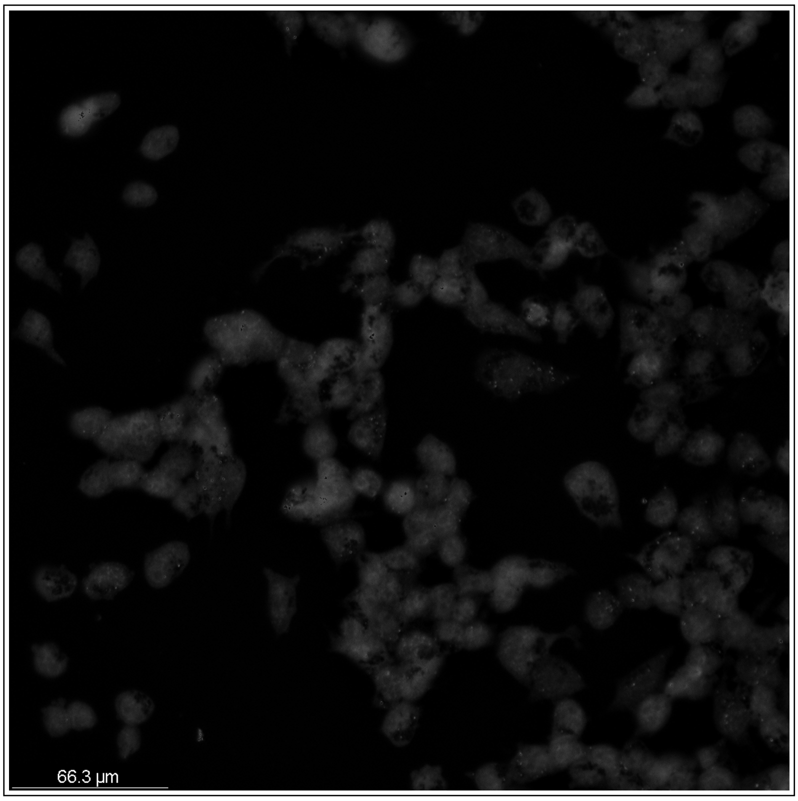

Channel 1
ptau396-16.tif

Channel 2 (Max Projection)
1024.0 x 1024.0

Channel 2
ptau396-16-drug.tif

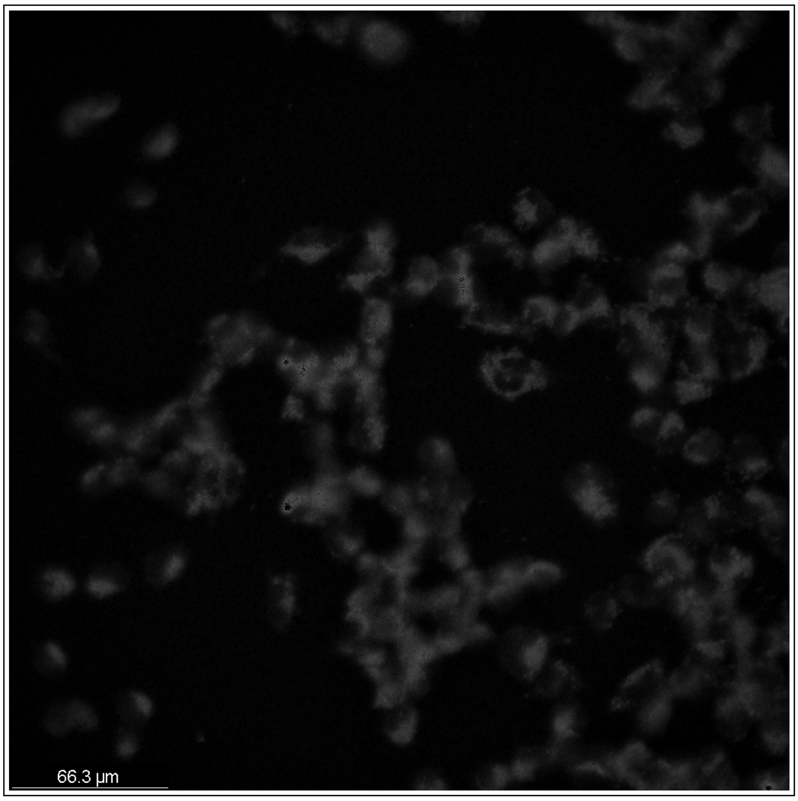

Channel 1
ptau396-16.tif

Warning! y-intercept far from zero - The ratio of the y-intercept of the auto threshold regression line to the mean value of Channel 2 is high. This means the y-intercept is far from zero, implying a significant positive or negative zero offset in the image data intensities. Maybe you should use a ROI. Maybe do a background subtraction in both channels. Make sure you didn't clip off the low intensities to zero. This might not affect Pearson's correlation values very much, but might harm other results.

Coloc_Job_Name: Colocalization_of_ptau396-16.tif_versus_ptau396-16-drug.tif_110273625

% zero-zero pixels: 4.09

% saturated ch1 pixels: 0.12

% saturated ch2 pixels: 0.12

Channel 1 Max: 255.000

Channel 2 Max: 255.000

Channel 1 Min: 0.000

Channel 2 Min: 0.000

Channel 1 Mean: 9.303

Channel 2 Mean: 11.134

Channel 1 Integrated (Sum) Intensity: 9755032.000

Channel 2 Integrated (Sum) Intensity: 11674757.000

Mask Type Used: none

Mask ID Used: 110273625

m (slope): 1.00

b (y-intercept): 1.81

b to y-mean ratio: 0.16

Ch1 Max Threshold: 3.00

Ch2 Max Threshold: 5.00

Threshold regression: Costes

Pearson's R value (no threshold): 0.74

Pearson's R value (below threshold): -0.04

Pearson's R value (above threshold): 0.63

Li's ICQ value: 0.401

Spearman's rank correlation value: 0.68997306

Spearman's correlation t-statistic: 976.0950

t-statistic degrees of freedom: 1048574.000

Manders' M1 (Above zero intensity of Ch2): 0.985

Manders' M2 (Above zero intensity of Ch1): 0.946

Manders' tM1 (Above autothreshold of Ch2): 0.928

Manders' tM2 (Above autothreshold of Ch1): 0.806

Kendall's Tau-b rank correlation value: 0.5207

Costes P-Value: 1.00

Costes Shuffled Mean: 0.00

Costes Shuffled Std.D.: 0.01

Ratio of rand. Pearsons >= actual Pearsons value : 0.00
