# Supplementary material for: Near‐Infrared Fluorescent PROTAC Enables Theranostic Imaging and Selective Tau Degradation in Alzheimer's Disease
Source: Adv Sci (Weinh). 2026 Sep 1:e77545. Online ahead of print. doi: 10.1002/advs.77545 (PMC13534785; doi:10.1002/advs.77545)
Supplement: Supplementary file 2 [file ADVS-9999-e77545-s001.zip › ptau404-0.53.pdf]

Colocalization_of_ptau404-16.tif_versus_ptau404-16-drug.tif_1732932541.pdf

Li - Ch1

256.0 x 256.0

Channel 2
ptau404-16-drug.tif

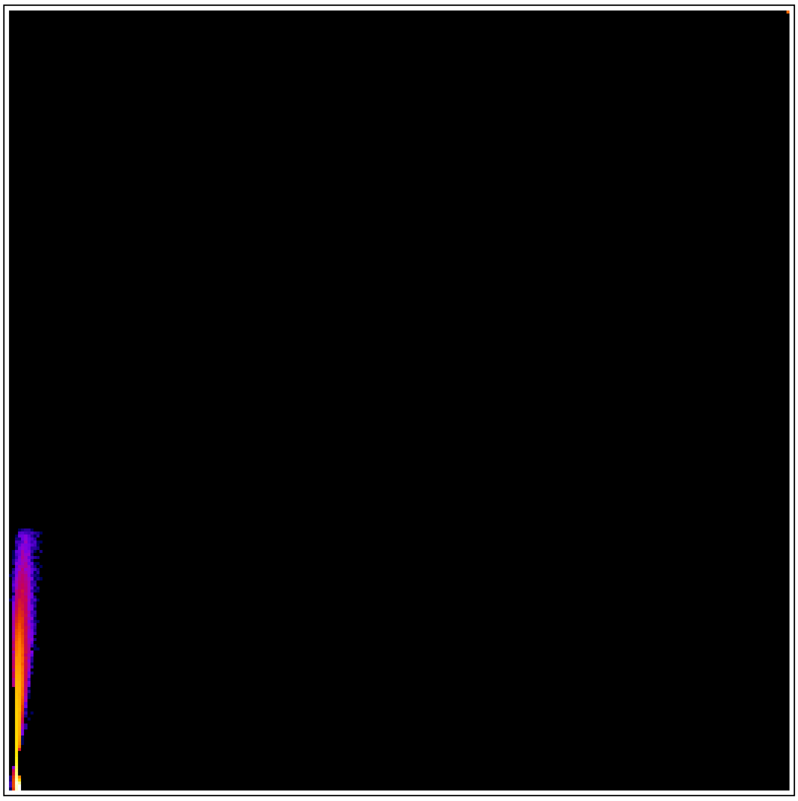

Channel 1
ptau404-16.tif

Li - Ch2

256.0 x 256.0

Channel 2
ptau404-16-drug.tif

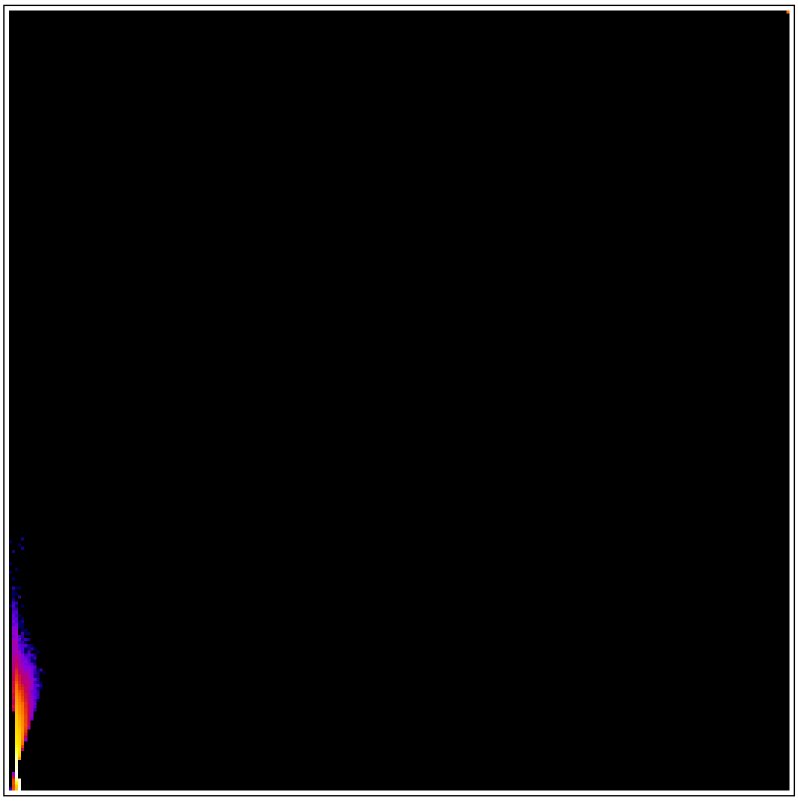

Channel 1
ptau404-16.tif

2D intensity histogram

256.0 x 256.0

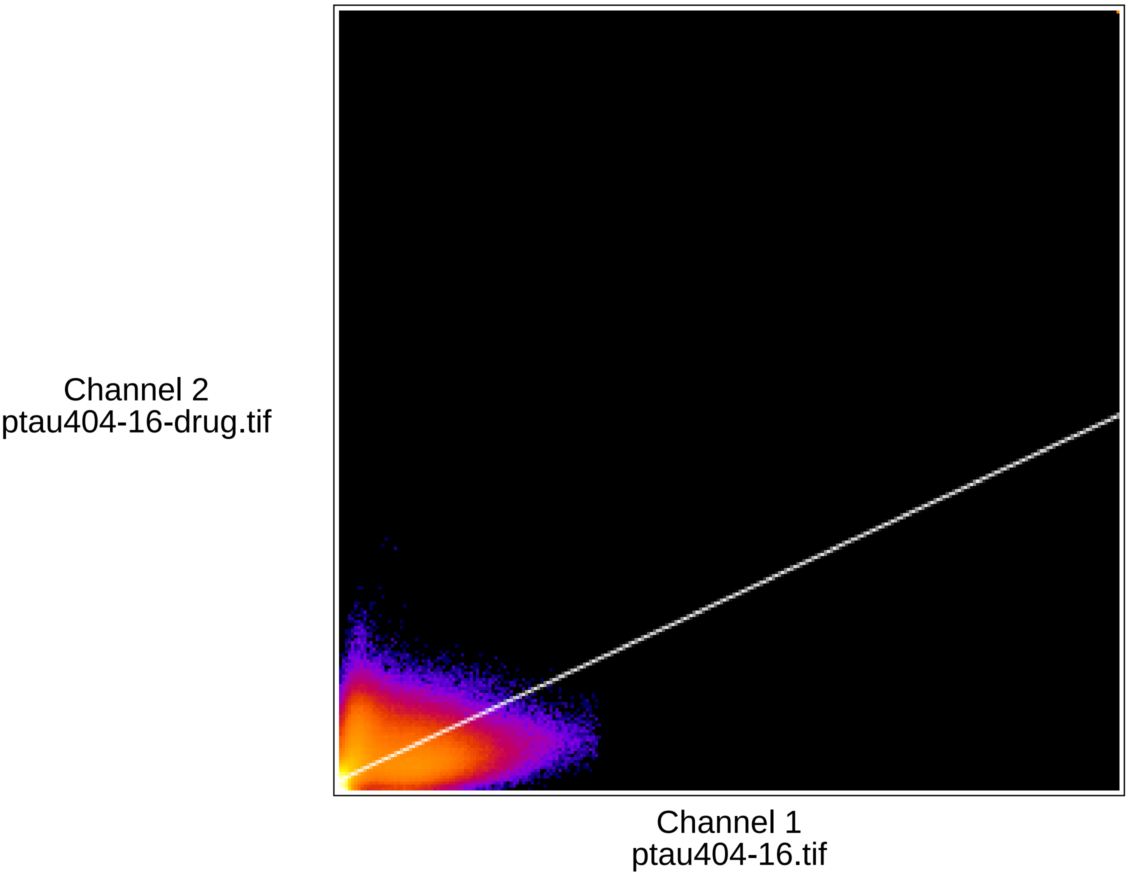

Smoothed & shuffled channel 1

1024.0 x 1024.0

Channel 2
ptau404-16-drug.tif

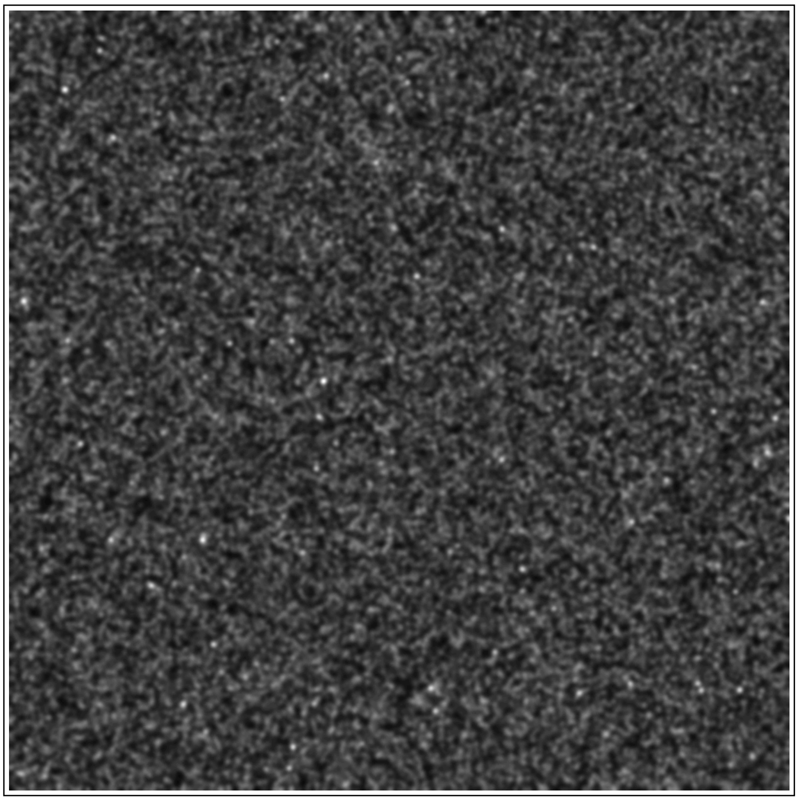

Channel 1
ptau404-16.tif

Channel 1 (Max Projection)

1024.0 x 1024.0

Channel 2
ptau404-16-drug.tif

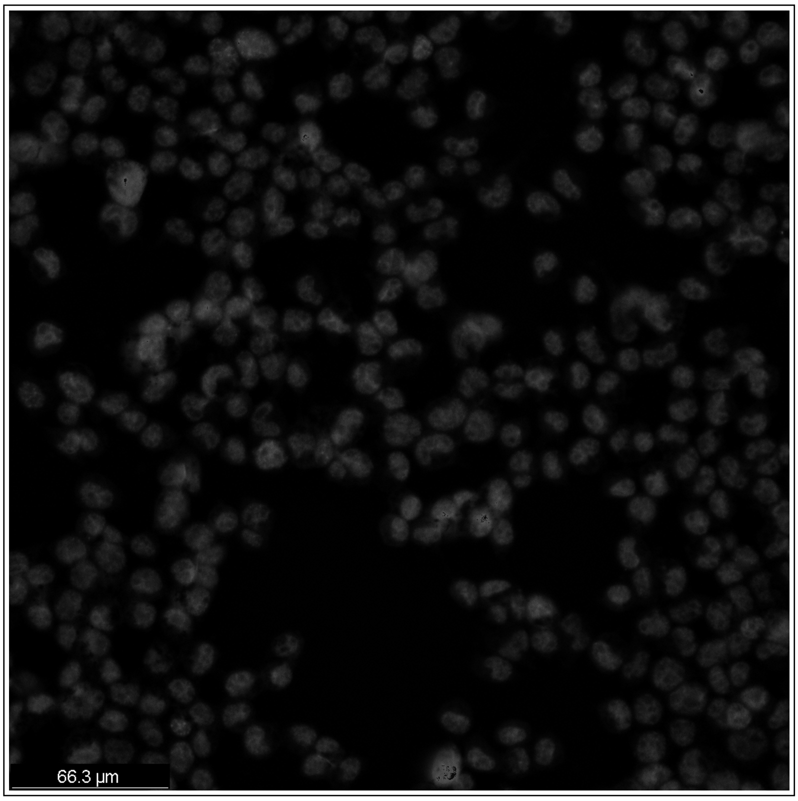

Channel 1
ptau404-16.tif

Channel 2 (Max Projection)

1024.0 x 1024.0

Channel 2
ptau404-16-drug.tif

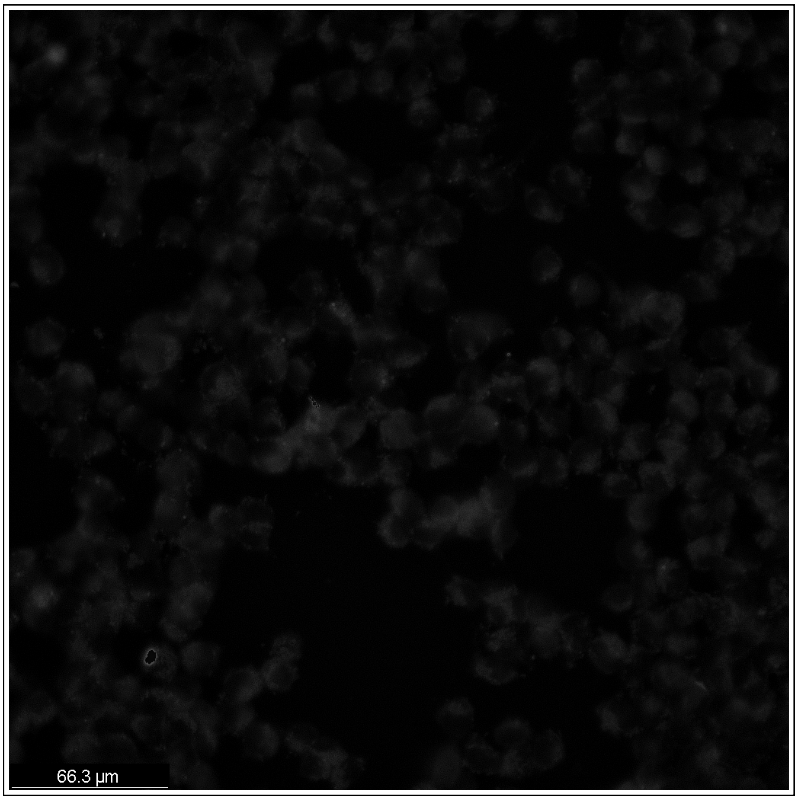

Channel 1
ptau404-16.tif

Warning! y-intercept far from zero - The ratio of the y-intercept of the auto threshold regression line to the mean value of Channel 2 is high. This means the y-intercept is far from zero, implying a significant positive or negative zero offset in the image data intensities. Maybe you should use a ROI. Maybe do a background subtraction in both channels. Make sure you didn't clip off the low intensities to zero. This might not affect Pearson's correlation values very much, but might harm other results.

Coloc_Job_Name: Colocalization_of_ptau404-16.tif_versus_ptau404-16-drug.tif_1732932541

% zero-zero pixels: 5.05

% saturated ch1 pixels: 0.12

% saturated ch2 pixels: 0.12

Channel 1 Max: 255.000

Channel 2 Max: 255.000

Channel 1 Min: 0.000

Channel 2 Min: 0.000

Channel 1 Mean: 10.969

Channel 2 Mean: 8.387

Channel 1 Integrated (Sum) Intensity: 11502319.000

Channel 2 Integrated (Sum) Intensity: 8794865.000

Mask Type Used: none

Mask ID Used: 1732932541

m (slope): 0.47

b (y-intercept): 3.25

b to y-mean ratio: 0.39
Ch1 Max Threshold: 4.00
Ch2 Max Threshold: 5.00
Threshold regression: Costes
Pearson's R value (no threshold): 0.53
Pearson's R value (below threshold): -0.02
Pearson's R value (above threshold): 0.36
Li's ICQ value: 0.215
Spearman's rank correlation value: 0.61751321
Spearman's correlation t-statistic: 803.9215
t-statistic degrees of freedom: 1048574.000
Manders' M1 (Above zero intensity of Ch2): 0.984
Manders' M2 (Above zero intensity of Ch1): 0.945
Manders' tM1 (Above autothreshold of Ch2): 0.877
Manders' tM2 (Above autothreshold of Ch1): 0.785
Kendall's Tau-b rank correlation value: 0.4426
Costes P-Value: 1.00
Costes Shuffled Mean: -0.00
Costes Shuffled Std.D.: 0.01
Ratio of rand. Pearsons >= actual Pearsons value : 0.00
